# Unravelling the specificity and mechanism of sialic acid recognition by the gut symbiont *Ruminococcus gnavus*

**DOI:** 10.1038/s41467-017-02109-8

**Published:** 2017-12-19

**Authors:** C. David Owen, Louise E. Tailford, Serena Monaco, Tanja Šuligoj, Laura Vaux, Romane Lallement, Zahra Khedri, Hai Yu, Karine Lecointe, John Walshaw, Sandra Tribolo, Marc Horrex, Andrew Bell, Xi Chen, Gary L. Taylor, Ajit Varki, Jesus Angulo, Nathalie Juge

**Affiliations:** 1 Biomolecular Sciences Building, University of St Andrews, St Andrews, KY16 9ST UK; 2 The Gut Health and Food Safety Programme, Quadram Institute Bioscience, Norwich Research Park, Norwich, NR4 7UA UK; 3 School of Pharmacy, University of East Anglia, Norwich Research Park, Norwich, NR4 7TJ UK; 4 Glycobiology Research and Training Center (GRTC), Departments of Medicine and Cellular and Molecular Medicine, UC San Diego, La Jolla, CA 92093-0687 USA; 5 Department of Chemistry, University of California-Davis, Davis, CA 95616 USA; 6 School of Computing Sciences, University of East Anglia, Norwich, NR4 7TJ UK; 7 Present Address: Diamond Light Source Ltd, Diamond House, Harwell Science and Innovation Campus, Didcot, OX11 0FA UK; 8 Present Address: Ajinomoto Althea Inc, 11040 Roselle Street, San Diego, CA 92121 USA

## Abstract

*Ruminococcus gnavus* is a human gut symbiont wherein the ability to degrade mucins is mediated by an intramolecular *trans*-sialidase (*Rg*NanH). *Rg*NanH comprises a GH33 catalytic domain and a sialic acid-binding carbohydrate-binding module (CBM40). Here we used glycan arrays, STD NMR, X-ray crystallography, mutagenesis and binding assays to determine the structure and function of *Rg*NanH_CBM40 (*Rg*CBM40). *Rg*CBM40 displays the canonical CBM40 β-sandwich fold and broad specificity towards sialoglycans with millimolar binding affinity towards α2,3- or α2,6-sialyllactose. *Rg*CBM40 binds to mucus produced by goblet cells and to purified mucins, providing direct evidence for a CBM40 as a novel bacterial mucus adhesin. Bioinformatics data show that *Rg*CBM40 canonical type domains are widespread among Firmicutes. Furthermore, binding of *R. gnavus* ATCC 29149 to intestinal mucus is sialic acid mediated. Together, this study reveals novel features of CBMs which may contribute to the biogeography of symbiotic bacteria in the gut.

## Abstract (web summary)

The mucus layer is an important physical niche within the gut which harbours a distinct microbial community. Here the authors show that specific carbohydrate-binding modules associated with bacterial carbohydrate-active enzymes are mucus adhesins that target regions of the distal colon rich in sialomucins.

## Introduction

The human gut microbiota encompasses a complex community of bacterial species, which play a critical role in human health, through their contribution to e.g., polysaccharide digestion, immune system development and pathogen defence^1^. Microbiota composition varies longitudinally along the gastrointestinal (GI) tract but also transversally from the lumen to the mucosa^1,2^. Most gut bacteria reside in the colon, reaching 10^11^–10^12^ cells per gram, where they compete for dietary and host glycans^3,4^. A dysbiosis of the gut microbiota is associated with intestinal diseases, including cancers, infections and inflammatory bowel diseases^5–8^, underscoring the importance of understanding these host-microbe interactions in order to devise novel treatment strategies.

Several factors influence the biogeography of symbiotic bacteria within the gut, including the gradient and availability of glycans within discrete physical niches^2,3^. The mucus layer covering the GI tract is at the interface between the gut microbiota and the host^5^. In the colon, the mucus layer is divided into a loose outer layer providing a habitat to commensal bacteria and an inner layer adhering to the epithelium and providing protection from bacterial invasion^5^. The outer mucus layer hosts a distinct intestinal microbial niche^9^. The intestinal mucus layers are built around large highly glycosylated gel-forming mucin MUC2 (Muc2 in mouse) secreted by goblet cells^10^. The glycan structures present in mucins are diverse and complex and consist of four core mucin-type *O*-glycans containing *N*-acetylgalactosamine (GalNAc), galactose (Gal) and *N*-acetylglucosamine (GlcNAc). Mucin *O*-glycosylation starts with the attachment of GalNAc residues to the hydroxyl group of Ser and Thr of the protein backbone to form the Tn antigen (GalNAcα1-Ser/Thr). This glycan is then elongated into core 1 (Galβ1-3GalNAcα1-Ser/Thr, also known as Thomsen Friedenreich-TF- or T-antigen), core 2 (Galβ1-3(GlcNAcβ1-6)GalNAcα1-Ser/Thr), core 3 (GlcNAcβ1-3GalNAcα1-Ser/Thr) or core 4 (GlcNAcβ1-3(GlcNAcβ1-6)GalNAcα1-Ser/Thr)^11^. Core 3-derived *O*-glycans are important components of human colonic mucin-type *O*-glycans^12^. These core structures are further elongated by the addition of other carbohydrates (e.g., *N*-acetyllactosamine, LacNAc) and are most commonly terminated by fucose and sialic acid sugar residues via α1–2/3/4 and α2–3/6 linkages, respectively. These oligosaccharide chains provide binding sites and nutrients to the bacteria which have adapted to the mucosal environment^13,14^. Reflecting the structural diversity of mucin glycans and their prime location, commensal and pathogenic microbes have evolved a range of adhesins allowing their interaction with mucus^13,15^. Variation in mucosal carbohydrate availability leads to variations in the composition of the resident microbiota^3,16,17^ and may also impact on bacterial tropism along and across the GI tract^18^.

Sialic acids, such as *N*-acetylneuraminic acid (Neu5Ac) and fucose residues in terminating positions on mucin glycan chains are prominent targets for commensal and pathogenic bacteria^19,20^. The ratio of sialic acid to fucose increases along the GI tract, from the ileum to the rectum in humans^21^ and an inverse gradient occurs in mice^22^. Furthermore blood group Sd^a^/Cad related epitopes, GalNAcβ1-4(NeuAcα2-3)Gal, increase along the length of the human colon^12^. Over 100 complex oligosaccharides can be identified in mucins from human colonic biopsies, with most being mono-, di- or trisialylated^23^. Release of sialic acid by microbial sialidases allows bacteria to access free sialic acid for catabolism, decrypt host ligands for adherence, participate in biofilm formation, modulate immune function by metabolic incorporation, and expose the underlying glycans for further degradation^10,14,19,20^. Sialidases are often associated with additional domains including carbohydrate binding modules (CBMs), such as sialic acid-specific CBM40^14,24^ and broadly specific CBM32^25^. CBMs can enhance catalytic activity by concentrating the enzymes onto carbohydrate substrates^26^ or mediate adherence to host cells^27^.

*Ruminococcus gnavus* is a prominent member of the gut microbiota of the healthy human gut^28^. *R. gnavus* utilisation of mucin is associated with the expression of an intramolecular *trans*-sialidase (IT-sialidase)^29,30^, which is proposed to play a key role in the adaptation of gut bacteria to the mucosal environment by providing 2,7-anhydro-sialic acid as a preferential source of nutrients^31^. The IT-sialidase from *R. gnavus* (*Rg*) ATCC 29149 (*Rg*NanH) comprises a catalytic glycoside hydrolase domain, *Rg*GH33 and a carbohydrate binding module, *Rg*CBM40.

Here, to gain insights into the role and specificity of sialic acid recognition by *R. gnavus*, we employed glycan microarray, X-ray crystallography, saturation transfer difference nuclear magnetic resonance spectroscopy (STD NMR), isothermal titration calorimetry (ITC), mutational analyses, and cell/tissue binding assays to identify *Rg*CBM40 oligosaccharide binding partners. Prominent ligands were oligosaccharides with terminal sialic acid, including those which are not substrates for *Rg*NanH activity. We propose a novel role for CBM40 in targeting gut bacteria towards sialic acid-rich regions of the GI tract.

## Results

### *Rg*CBM40 belongs to the CBM40 subfamily

**Fig. 1:**
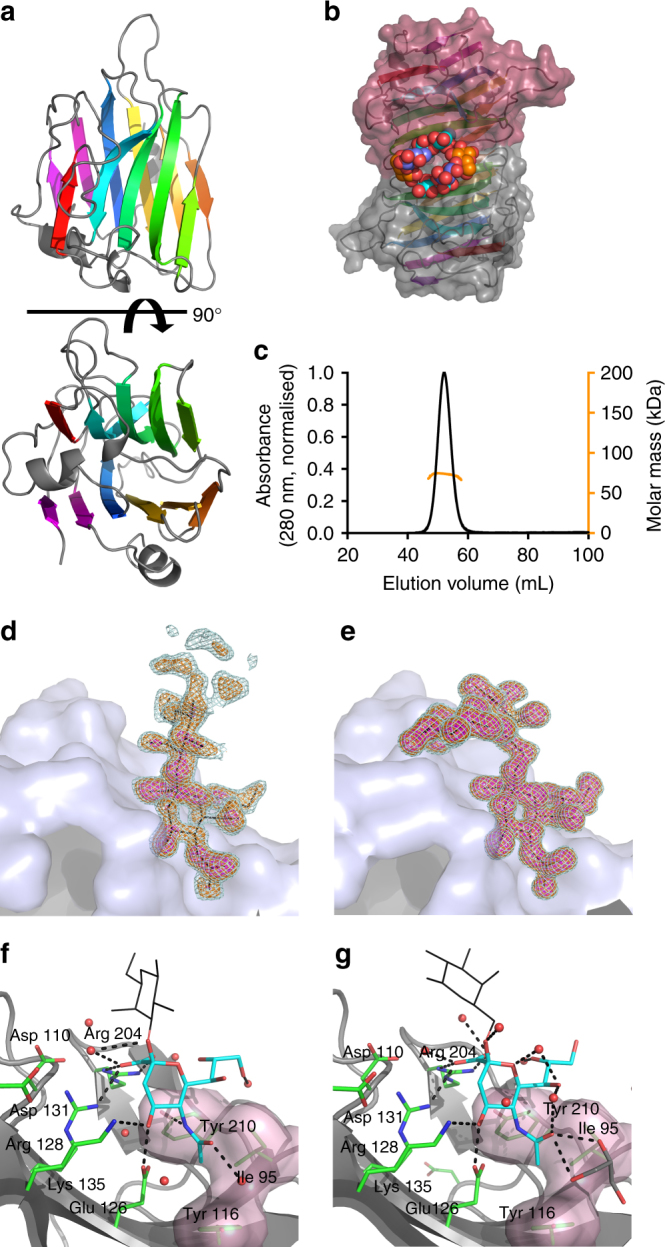
Crystal structure of *Rg*CBM40 in complex with 3′SL and 6′SL. **a** *Rg*CBM40 is shown in a cartoon representation with a rotation of 90° around the *x* axis. **b** The protein crystallised as a dimer with the ligand binding site at the dimer interface. The binding sites are shown occupied by 6′SL trisaccharides (Neu5Ac: cyan, galactose: blue, glucose: orange). **c** SEC-MALS performed with full length *Rg*NanH (77 kDa). The SEC-MALS predicted molecular weight was 73 kDa, indicating that *Rg*NanH is monomeric in solution. Bound (**d**) 3′SL and (**e**) 6′SL are shown with their corresponding Fo-Fc omit maps at 2σ (light cyan), 3σ (orange) and 5σ (magenta). The omit maps are carved at 1.6 Å around the bound ligand. For 3′SL, the map is carved around a dummy glucose residue to indicate the presence of partial electron density. A close-up view of *Rg*CBM40 binding site is shown with (**f**) 3′SL and (**g**) 6′SL. The Neu5Ac residue is shown in cyan and the galactose residue as black lines, for clarity the glucose residue is not shown. Interacting *Rg*CBM40 residues are shown in green with black dashed lines indicating hydrogen bonding interactions. A semi-transparent surface indicates the hydrophobic surface

*Rg*CBM40 crystallised as a dimer, adopting the canonical CBM40 β-sandwich fold with six antiparallel strands on the convex face and five on the concave face (Fig. 1a, for data collection and refinement statistics, see Table 1). Electron density was observed for all *Rg*CBM40 residues present in the construct (50–237). The sialic acid binding site is on the concave face at the dimer interface (Fig. 1b), however, size exclusion chromatography with multi angle light scattering (SEC-MALS) indicated that the full-length protein, *Rg*NanH, is monomeric in solution (Fig. 1c). The macromolecular architecture of *Rg*CBM40 is conserved among members of the CBM40 family (Supplementary Fig. 1), with the exception of *Vibrio cholerae* CBM40_NanH (*Vc*CBM40_NanH) which is proposed to be part of a separate CBM40 subfamily (Supplementary Fig. 1h)^25,32^. Greatest structural homology was observed to *Md*CBM40 NanL (RMSD: 0.3 Å) from the *Macrobdella decora* IT-sialidase (Supplementary Fig. 1e)^33^.

**Table 1:** Data collection and refinement statistics

Data set	Apo	3′SL	6′SL
*Data collection*			
Space group	P21	P21	P21
*Cell dimensions*			
*a*, *b*, *c* (Å)	46.7, 72.8, 51.3	48.8, 72.4 51.5	48.7, 72.2, 51.4
*β* (°)	104.9	105.1	103.9
Resolution	49.56–1.73 (1.76–1.73)	39.48–1.37 (1.41–1.37)	49.91–1.30 (1.34–1.30)
*R*_merge_	0.03 (0.14)	0.04 (0.34)	0.03 (0.15)
*I*/*σI*	47.3 (9.6)	22.9 (3.0)	32.0 (4.9)
Completeness	91.8 (51.3)	74.5 (11.2)	83.9 (13.6)
Redundancy	3.7 (2.4)	4.3 (2.4)	5.1 (1.4)
*Refinement*			
Resolution	49.56–1.73 (1.76–1.73)	39.48–1.37 (1.41–1.37)	49.91–1.30 (1.34–1.30)
No. of reflections	31,570	51,221	67,097
*R*_work_/*R*_free_	0.160/0.194 (0.82)	0.152/0.187 (0.81)	0.134/0.154 (0.87)
No. of atoms	3145	3424	3704
Protein	2807	2850	3076
Ligand	0	81	123
Water	338	508	527
*B*-factors			
Protein	19.4	16.6	10.4
Ligand/ion		36.4	21.7
Water	28.1	31.7	27.3
*R.m.s.d*			
Bond lengths (Å)	0.011	0.012	0.015
Bond angle (°)	1.55	1.66	1.77

Values in parentheses refer to the highest resolution shell. For the 3′SL and 6′SL complexes the data were over 90% complete to a resolution of 1.85 Å and 1.56 Å, respectively. One crystal was used for each structure

Protein ligand complexes were achieved for both 3′SL and 6′SL (Fig. 1d, e). No significant conformational changes were observed in the binding site upon ligand binding. Definitive electron density for the Neu5Ac and galactose residues was observed in the 3′SL and 6′SL complexes. In the 6′SL complex, electron density was also observed for the glucose residues (Fig. 1e), with the lactose positioned almost perpendicular to the sialic acid (Fig. 1e). Contrastingly, for the 3′SL complex, only partial electron density was observed for the glucose residue in a single monomer (Fig. 1d), and the glucose positioning indicates that the lactose points up and away from the binding site, without further interactions with the protein. In the 3′SL complex, the lactose positioning would permit further extensions to the carbohydrate chain as would be present in more complex or anchored glycans, whereas these may be blocked in the 6′SL complex. This would provide a degree of specificity towards sialic acid linkage.

Neu5Ac binds in a chair conformation (Fig. 1f, g), mimicking the solution conformation and minimising the energetic penalty paid upon binding^26^. Notably, the carboxylic acid group of Neu5Ac forms electrostatic interactions with an arginine dyad, Arg204 and Arg128, mimicking the coordination observed in sialidase active sites. The C4 hydroxyl group hydrogen bonds to Lys135 and Glu126, the *N*-acetyl group sits in a hydrophobic pocket formed by Tyr116 and Ile95. The *N*-acetyl group nitrogen interacts with both Glu126 and Tyr210. Glu126, Arg128 and Arg204 make extensive interactions with the bound ligand and are conserved in all structurally characterised CBM40 sialic acid binding sites, discounting *Vc*CBM40_NanH^34^ (Supplementary Fig. 2). The environment of the glycerol side-chain of sialic acid is generally conserved across the canonical CBM40 subfamily with the rear face (C7-H and C9-H groups) residing on a hydrophobic surface formed by Ile95 and Tyr210 in *Rg*CBM40 (Supplementary Fig. 3a). Although *Vc*CBM40_NanH shares the CBM40 β-sandwich fold (Supplementary Fig. 1), the location, orientation, and constitution of its sialic acid binding site is not conserved (Supplementary Fig. 2).

### Structure-based sequence alignment

**Fig. 2:**
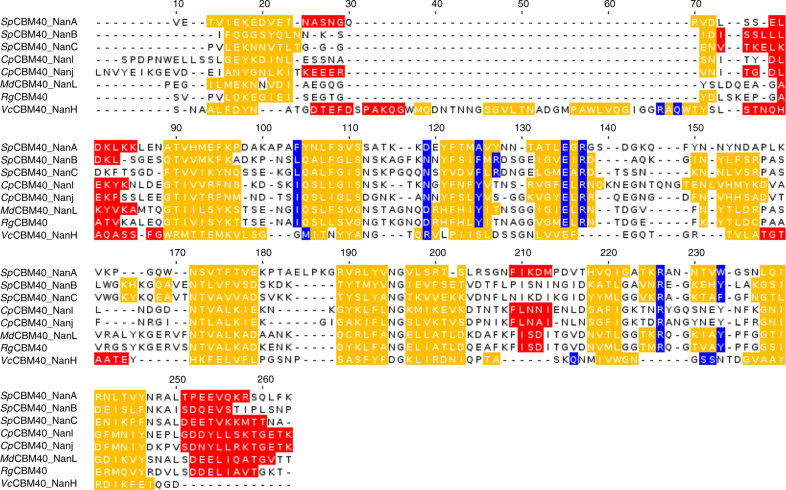
CBM40 structural alignment. Structure-based alignment (α-helices and β-strands respectively in red and yellow) of CBM40 domains of *Rg*CBM40 with *C. perfringens Cp*CBM40_NanJ (PDB code 2V73) and *Cp*CBM40_NanI (PDB code 5FRA), *M. decora Md*CBM40_NanL (PDB code 1SLI) and *S. pneumoniae Sp*CBM40_NanA (PDB code 4C1W), *Sp*CBM40_NanB (PDB code 2VW0) and *Sp*CBM40_NanC (PDB code 4YZ5) and *Vc*CBM40_NanH structure (PDB code 2W68). Amino acids identified as binding sites are highlighted in blue. *Rg*CBM40 residues Ile95, Asp110, Tyr116, Glu126, Arg128, Arg204 and Tyr210 are at positions 104, 119, 125, 135, 137, 226 and 233 of the alignment. The alignment supplemented with other canonical and *Vibrio*-type CBM40 sequences, used to create the pHMM using HMMER3, is shown in Supplementary Fig. 4

CBM40s associated with sialidases fall into two subfamilies, the canonical subfamily exemplified by *Cp*CBM40_NanJ^25^ (which also regroups *Rg*CBM40, *Cp*CBM40_NanI^32^, *Sp*CBM40_NanA^35^, *Sp*CBM40_NanB^36^, *Sp*CBM40_NanC^37^ and *Md*CBM40 NanL^33^), and the *Vibrio* subfamily exemplified by *Vc*CBM40_NanH^34^. Considerable sequence divergence between the *Vibrio* and canonical CBM40 types renders satisfactory alignments difficult to produce with standard tools, as also previously reported^32^. Here, by detailed manual inspection, paying particular attention to the limits of secondary structure elements and intervening loops, we produced an alignment of both types of CBM40 sequences showing well-conserved positions along its length, notwithstanding the *Vibrio* insertion (40 residues) near the N-terminus. The pairwise identities between the canonical representatives range from 21 to 67%, while the maximum canonical vs. *Vibrio* identity is 17%, reflecting that CBM40s fall into two distinct groups. This highlighted conserved residues within the canonical subfamily that may be involved in binding affinity and specificity (Fig. 2). These include (*Rg*CBM40 numbering): an arginine dyad (Arg204 and Arg128) that interacts with the sialic acid carboxylic acid group, a glutamic acid (Glu126), which hydrogen bonds to the C4 hydroxyl; and a hydrophobic surface, which accommodates the *N*-acetyl moiety and the hydrophobic face of the glycerol group. Tyr116, Ile95, Tyr210 contribute to the surface of an aromatic:aliphatic:aromatic twisted platform which presents the glycerol hydroxyl groups to solvent^26^.

### Bioinformatics analyses

**Fig. 3:**
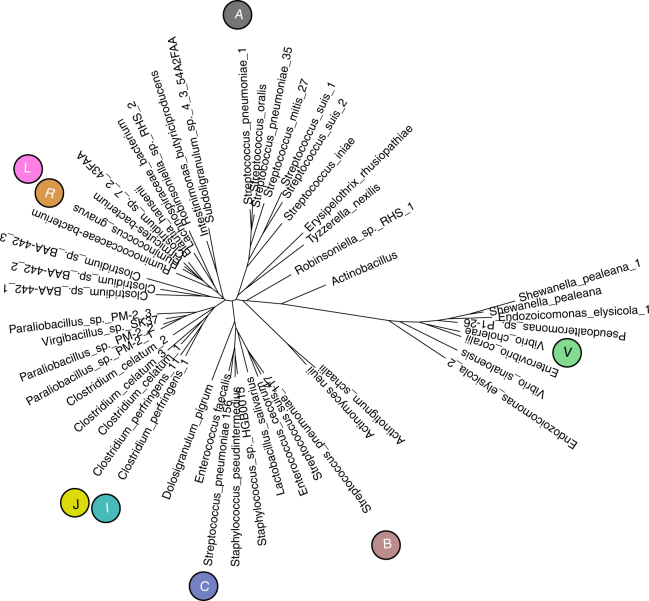
Distance-based tree of canonical and *Vibrio-*type CBM40 sequences. Tree of 51 non-redundant sequences (80% identity level) calculated by neighbour-joining using evolutionary distances estimated by applying the PMB model of amino acid changes, including all sites and using a uniform rate of evolution. The representative sequences corresponding most closely (at least 97% identical) to the 7 bacterial structure-determined sequences are shown with symbols, coloured in accordance with Supplementary Fig. 1: “A”, *Sp*CBM40_NanA; “B”, *Sp*CBM40_NanB; “C”, *Sp*CBM40_NanC; “I”, *Cp*CBM40_NanI; “J”, *Cp*CBM40_NanJ; “*R*”, *Rg*CBM40; “*V*”, *Vc*CBM40_NanH. Additionally, “L” denotes *Md*CBM40_NanL closest to the bacterial sequence of highest identity (70% identical to *Rg*CBM40) as only bacterial sequences were searched

To gain further insights into the phylotypic distribution of the CBM40 domains within bacterial genomes, we performed a database search using pHMMs derived from our alignment as queries (canonical and *Vibrio*-type together, referred to as ‘combined’; canonical only; *Vibrio-*type only) as well as Pfam models, “Sialidase(NTD)”, “Laminin_G_3”, and “Sial-lect-inser” (see Methods section and Supplementary Methods). Our combined model successfully identified 99.9% of the CBM40 domains matched by the individual type CBM40 models (over 16,000 domain hits in the whole database of around 67,000 genomes). Further analysis of the data (Supplementary Methods) led to the identification of 51 nonredundant sequences (Supplementary Fig. 4). Of these, the canonical CBM40 domains occurred in Firmicutes with 40 sequences, representing 18 genera or pseudogenera, divided between classes Bacilli and Clostridia, as well as Erysipelotrichi and an unclassified member of the Firmicutes; and two sequences in Actinobacteria. The *Vibrio*-type occurred only in Gammaproteobacteria, represented by 8 sequences in five genera. The separation between the *Vibrio*-type sequences and canonical CBM40 sequences across bacterial genomes was also apparent from a tree representation constructed using a simple distance-based model and neighbour-joining (Fig. 3). This dichotomy was fully supported by bootstrap analysis of 1000 replicates. There was no evidence for any intermediate or other CBM40 types. Only one sequence from *Actinobacillus muris* containing a canonical CBM40 (confirmed by pHMMs and conserved binding residues) was shown to be part of a Gammaproteobacteria clade (all other members *Vibrio*-type) as supported by 79% of bootstraps. Further studies may indicate whether this domain is the closest to an inferred common ancestor of the canonical and *Vibrio* CBM40 types. The results for co-incidence of sialidase domains clearly indicated an association with CBM40s in this set of non-redundant sequences: we detected a sialidase domain in 92% of canonical-type CBM40 and in all *Vibrio*-type CBM40 representatives.

### *Rg*CBM40 preferentially binds α2,3 linked sialosides

**Fig. 4:**
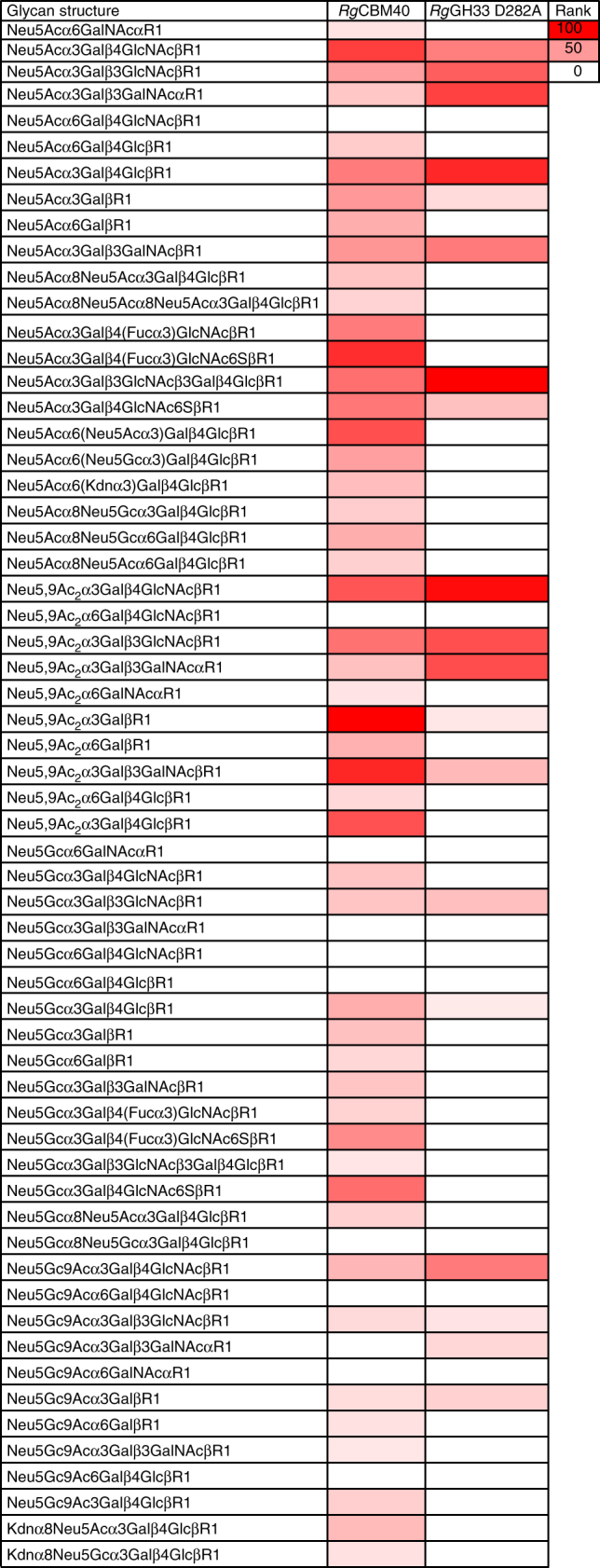
Sialoglycan microarray analysis of binding specificities of *Rg*CBM40 and *Rg*GH33 D282A. Binding of the recombinant proteins *Rg*CBM40 and *Rg*GH33 D282A at 20 and 200 µg ml^−1^, respectively are presented (*n* = 4, SD). Heat map was generated using the method as previously described^38,39^. Binding was ranked as (glycan average RFU/ maximum glycan average RFU)×100. Red and white represent the maximum and minimum, respectively. R1 represents propyl-azide as the spacer

To further explore *Rg*NanH ligand specificity, *Rg*CBM40 and inactive mutant *Rg*GH33 D282A, were tested for binding to various sialoglycans, using a slide microarray^38,39^. This sialoglycan microarray presents over 60 synthetically recreated naturally occurring oligosaccharide structures with diverse sialic acid forms, glycosidic linkages, and underlying glycans, representing a broad range of such targets^38,39^. Both recombinant proteins exclusively bound to glycans terminated with sialic acids (Fig. 4). They also showed distinct specificities. *Rg*CBM40 bound to most but not all glycans bearing terminal Neu5Ac, Neu5Gc, Neu5,9Ac_2_ and 2-keto-3 deoxynonulosonic acid (Kdn) attached with α2-3, α2-6 and α2-8 linkages, with weaker binding for Neu5Gc and kdn oligosaccharides (Fig. 4). In contrast, *Rg*GH33 D282A interacted weakly with a narrow spectrum of sialoglycans, mainly α2-3-Neu5Ac-containing glycans, primarily Neu5Acα3LacNAcβ (3′SLN), Neu5Acα3Galβ3GlcNAcβ, Neu5Acα3Galβ3GalNAc (STF), Neu5Acα3Lacβ (GM3) and Neu5Acα3Galβ3GalNAcβ3Lac (Fig. 4). Noticeably, *Rg*GH33 D282A recognised some of the α2-3-linked sialoglycans but not any α2-6- or α2-8-linked ones, in line with its substrate specificity^30^. In marked contrast, every α2-3-linked sialyl oligosaccharide present on the array could be bound by *Rg*CBM40. *Rg*CBM40 showed a preference for terminal Neu5Ac over Neu5Gc, and for α2-3≫α2-6 > α2-8 linkages. *Rg*CBM40 bound generally more strongly to glycans containing LacNAc and Lac. *Rg*CBM40 could bind Neu5Ac linked Lac with α2-3 and α2-6 linkage, albeit to a lesser degree, whereas binding to Neu5Ac linked LacNAc was α2-3-specific. Due to the glycan orientation introduced by the α2-6-sialic acid linkage the 6′SL glucose residue is close to the protein surface (Fig. 1e). Therefore, α2-6-linked LacNAc *N*-acetyl group may be blocked by protein residues, whereas the α2-3 linked glycan would be more solvent exposed. The highest binding was to Neu5,9Ac_2_α3GalβR1. Interestingly, *Rg*CBM40 bound to Neu5Gcα3Galβ3GalNAcβR1 (Neu5Gc-TF) and Neu5Gc9Acα3Galβ3GalNAcβR1 (Neu5Gc9Ac-TF) although with 5–10-fold less intensity, but it could not bind to the same ligands with the αR1 linkage. *Rg*CBM40 bound to α2-3-sialylated Lewis X (3′SLX, both Neu5Ac and Neu5Gc forms, although Neu5Ac was preferred). Sulfation of the 6 position of GlcNAc in 3′SLX (both Neu5Ac and Neu5Gc) improved binding of the protein (Fig. 4).

To validate some of the glycan array data, we used STD NMR spectroscopy^40,41^ against a range of sialylated ligands. Since the highest STD intensities correlate with the closest ligand-protein contacts in the bound state^42^, STD NMR experiments provide important information on the binding epitope of the complexed ligand^43^.

**Fig. 5:**
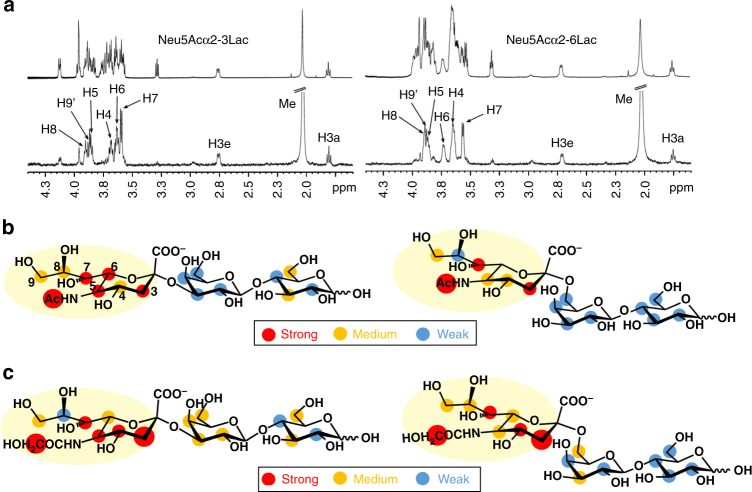
STD NMR analysis of *Rg*CBM40 binding to sialoglycans. **a** Reference (top) and difference (bottom) spectra of 3′SL and 6′SL. The strongest signals from the Neu5Ac’s protons are labelled in the difference spectra. **b** Binding epitope mapping from STD NMR of 3′SL and 6′SL. Legend indicates relative STD intensities normalised at H7: blue, 0–24%; yellow, 25–50%; red 51–100%; larger red dots indicate values over 100%. Sialic acid is the main recognition element. **c** Binding epitopes mapping from STD NMR of Neu5Gcα2-3Lac and Neu5Gcα2-6Lac. Legend as above. Sialic acid is the main recognition element. The strongest STD intensities from CH2 and the H3s, suggest a reorientation of the Neu5Gc ring in the binding pocket, in comparison to 3′SL and 6′SL

Here Neu5Ac, Neu5Gc, 2,7-anhydro-Neu5Ac, 3′SL, 6′SL, Neu5Acα3Gal (3′SGal), Neu5Acα6 Gal (6′SGal), 3′SLN, Neu5Acα6LacNAc (6′SLN), Neu5Gcα3Lac (3′SLGc), Neu5Gcα6Lac (6′SLGc), Neu5Acα6GalαOC3H6N3 (Neu5Ac-STn), Neu5Gcα6GalαOC3H6N3 (Neu5Gc-STn) and STFαOC3H6N3 were tested as potential ligands for *Rg*CBM40. With the exception of the three monosaccharides, Neu5Ac, Neu5Gc and 2,7-anhydro-Neu5Ac, binding to *Rg*CBM40 was detected for all di- and trisaccharides tested. For the latter, the binding epitope mapping was obtained and analysed as described under Methods section. Figure 5a shows the STD NMR spectra of 3′SL and 6′SL, and Fig. 5b their binding epitope mapping. The sialic acid ring was found to be the main recognition element and the binding mode was not affected by the nature of the glycosidic linkage (α2-3 or α2-6) of the sialoglycan (Supplementary Fig. 5). The same was true for the other Neu5Ac-ending ligands tested (see binding epitope mapping in Supplementary Fig. 6). The overall binding epitopes of 3′SL and 6′SL from the STD NMR in solution state are in good agreement with the crystal structures (Fig. 5), where the sialic acid is in close contact to the protein surface while the lactose moiety is solvent exposed as suggested from the very low STD intensities observed for the galactose and glucose protons. Very strong STD intensity were observed at the methyl group (Fig. 5). This is in excellent agreement with the *N*-acetyl group sitting in the hydrophobic pocket facing many protein protons (Hδ and Hγ) from the side chains of Ile95, Tyr116, and Tyr210 (Fig. 1f, g). High intensity on H7 compared to the much lower one on the adjacent H8 agrees with H7 facing the hydrophobic side chains while H8 (Fig. 5), in trans-conformation to it, is pointing towards the solvent. Within experimental error, no stark differences were observed in the orientation of the sialic acid ring in the binding pocket of *Rg*CBM40. *Rg*CBM40 also showed binding to Neu5Gc-ending oligosaccharides, albeit with a lower strength. Figure 5c shows the binding epitope of 3′SLGc and 6′SLGc (STD spectra are shown in Supplementary Fig. 6). Again, sialic acid was the main recognition element of these sialoglycans, but the binding epitope mapping was slightly different, in comparison to those of 3′SL and 6′SL. For the Neu5Gc-ending ligands, stronger STD intensities on H3s and lower ones on H6 were observed, suggesting a small reorientation of the ring around C3, which would expose C6, in order to fit the bulkier hydroxyl group on the acetamide moiety.

**Fig. 6:**
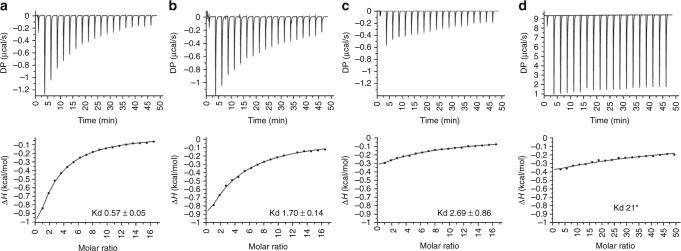
ITC isotherms of *Rg*CBM40 to sialoglycans. **a** *Rg*CBM40 binding to 3′SL, **b** *Rg*CBM40 binding to 6′SL, **c** *Rg*CBM40 binding to 3′SLGc, **d** *Rg*CBM40 binding to Neu5Ac. The Kd is indicated in mM. *This value is an estimate as the Kd is too high to determine with the concentration of sugar used

The affinity of the interaction between *Rg*CBM40 and sialic acid ligands was further assessed by ITC. Both 3′SL and 6′SL bound with similar low affinities, with dissociation constants of 0.57 mM and 1.70 mM, respectively (Fig. 6a, b, Supplementary Table 1). This confirms that *Rg*CBM40 is specific for the terminal residue irrespective of the glycosidic linkage but with a slight preference (~three fold) for the 2–3 linkage. Furthermore, it would suggest that the additional binding interactions observed in the crystal structure of the complex between *Rg*CBM40 and 6′SL do not significantly promote binding, also in agreement with the STD NMR results, showing that sialic acid is the main binding epitope in solution. We confirmed that *Rg*CBM40 binds to Neu5Gc-oligosaccharides, albeit with lower affinity, in accordance with the glycan array and STD NMR results. *Rg*CBM40 has a Kd of ~3 mM and >10 mM towards 3′SLGc and 6′SLGc, respectively (Fig. 6c, Supplementary Table 1). Very weak (~20 mM) interaction was observed between *Rg*CBM40 and Neu5Ac (Fig. 6d) or Neu5Gc monosaccharides (Supplementary Table 1). The STD NMR experiments were carried out with 1 mM sugar, well below the Kd, which explains why no interaction was observed using this approach. Thermodynamic analysis showed that the reaction is enthalpy-driven (Supplementary Table 2).

To further assess the involvement of individual residues, we introduced point mutations specifically designed to abrogate CBM binding. Arg128, Arg204, Tyr116, Tyr210, Glu126 and Ile95 were chosen for alanine substitutions. Analysis of the secondary structure by circular dichroism (CD) suggests that the recombinant proteins were correctly folded (Supplementary Fig. 7). Binding to Neu5Ac, 3′SL and 6′SL was abolished for the double mutant R128A/R204A as well as all single mutants, with the exception of I95A as shown by ITC (Supplementary Fig. 8a, b, Supplementary Table 1). I95A binds 3′SL and 6′SL with a Kd of 1.82 and 1.37 mM, respectively, broadly similar to the binding of the wild-type enzyme (Supplementary Table 1). This suggests that Ile95 is not an essential component of the hydrophobic pocket or the aromatic:aliphatic:aromatic twisted platform, and that the Tyr residues may compensate for the mutation of Ile95 to Ala. The binding ability of I95A to 3′SL and 6′SL was further confirmed by STD-NMR (Supplementary Fig. 9).

Taken together, the STD NMR and ITC data confirmed binding of both α2-3 and α2-6 linked sugars and raise questions regarding differences in ligand specificity between the catalytic and carbohydrate binding domains constituting *Rg*NanH. We previously showed that *Rg*NanH is specific for α2-3-linked substrates^30^. To determine the influence of *Rg*CBM40 on the sialidase activity, we compared the enzymatic activity of *Rg*NanH and *Rg*GH33 on a range of sialylated substrates. The reaction was monitored by HPAEC-PAD and showed no difference in catalytic activity on short oligosaccharides 3′SL, 3′SLX (Neu5Ac form) or on large polymeric MUC2 mucins (Supplementary Fig. 10), indicating that, in the conditions tested, *Rg*CBM40 did not potentiate the enzyme activity on these substrates.

### *Rg*CBM40 is a novel bacterial mucus adhesin

**Fig. 7:**
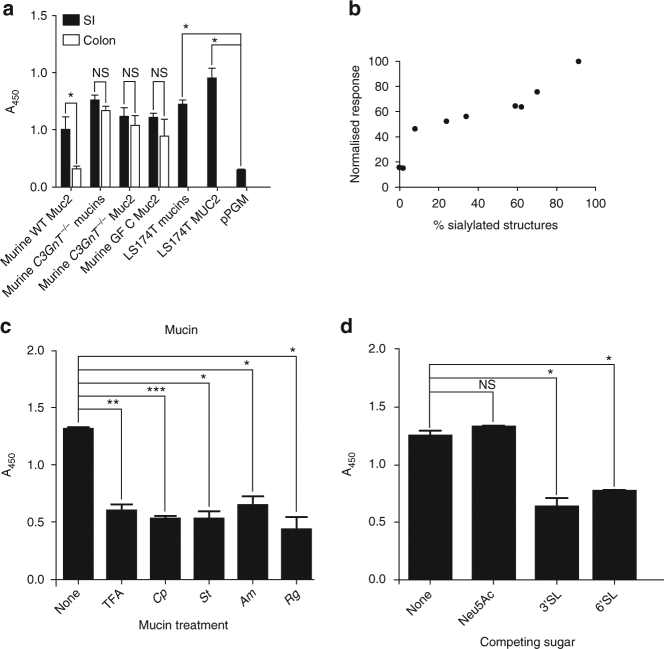
ELISA of *Rg*CBM40 binding to purified mucins. **a** *Rg*CBM40 binding to a range of purified mucins; mucin 2 (MUC2) and mixed mucins (mucins) from human cell line LS174T, purified pig gastric mucin (pPGM), and murine mucins from germ free (GF), wild type (WT), and *C3GnT* ^*−/−*^ mice. **b** Correlation of *Rg*CBM40 binding with % sialylated structure for each mucin tested. The % sialylated structures was determined by MS. **c** *Rg*CBM40 binding to LS174T MUC2 which has been treated chemically (TFA) or enzymatically with a sialidase from *Clostridium perfringens* (*Cp*), *Salmonella typhimurium* (*St*), *Akkermansia muciniphila* (*Am*) or *Ruminococcus gnavus* (*Rg*) **d** *Rg*CBM40 binding to LS174T MUC2 in competition with sugars. *Rg*CBM40 has been preincubated with the indicated sugars. In all cases, *Rg*CBM40 was incubated with immobilised mucins and binding detected using an anti-sialidase primary antibody and an anti-rabbit secondary antibody conjugated to horseradish peroxidase. The enzyme was incubated with TMB and the absorbance at 450 nm (A450) measured. The error bars show the standard error of the mean (SEM) of three replicates. *P* values are indicated; NS-not significant, *p < 0.05, **p < 0.005, ***p < 0.0005

*R. gnavus* ATCC 29149 but not the E1 strain encodes the IT-sialidase required for mucin-degradation^29,30^. Immunogold labelling and western blotting confirmed the presence of *Rg*NanH on *R. gnavus* ATCC 29149 cell-surface but not E1 (Supplementary Fig. 11a, b). Given the role of *Rg*NanH in *R. gnavus* mucin glycan utilisation, the binding of *Rg*CBM40 was tested by ELISA towards a range of mucins with different glycosylation profiles. The sialylation level of purified commercial pig gastric mucin (pPGM), mixed and Muc2/MUC2 mucins from mice and LS174T human cell line was analysed by mass spectrometry (MS), revealing that most of the mucins tested contained >8% sialylated structures; pPGM and Muc2 from the colon of wild-type C57BL/6 mice contained < 2% sialylated structures whereas the level of sialylation of LS174T MUC2 reaches 91% (Supplementary Table 3). Highest binding was observed to LS174T MUC2 whereas binding was lowest to pPGM or Muc2 from the colon of wild-type mice, which contain low levels of sialylation (Fig. 7a). The interaction was dependent on the concentration of *Rg*CBM40 (Supplementary Fig. 12). *Rg*CBM40 generally bound more strongly to mucins extracted from *C3GnT* ^*−/−*^ mice (mutants which lack core 3 β1-3-*N*-acetylglucosaminyltransferase, C3GnT)^44^ than to mucins from wild-type mice. Irrespective of the mouse model, the binding of *Rg*CBM40 to Muc2 from the small intestine was higher than from the colon (Fig. 7a). The adhesion level correlated well with the level of sialylation between the different mucins tested (*r* ^2^ = 0.88; Fig. 7b). *Rg*CBM40 bound significantly less strongly to MUC2 which has been treated with trifluoroacetic acid (TFA) to remove sialic acid, or with any of the sialidases tested which included the broad-specificity sialidase from *Clostridium perfringens* (*Cp*) and the α2-3-specific sialidases from *Salmonella typhimurium* (*St*), *Akkermansia muciniphila* (*Ak*) and *R. gnavus* (*Rg*), confirming the specificity of *Rg*CBM40 for terminal sialic acid (Fig. 7c). Consistent with the low affinity of CBM40 for Neu5Ac, this monosaccharide had no effect on adherence of *Rg*CBM40 to mucin (Fig. 7d). However, addition of free 3′SL or 6′SL prior to binding significantly decreased adherence of *Rg*CBM40 to MUC2 (Fig. 7d). These data indicate that *Rg*CBM40 recognises sialylated mammalian mucins.

**Fig. 8:**
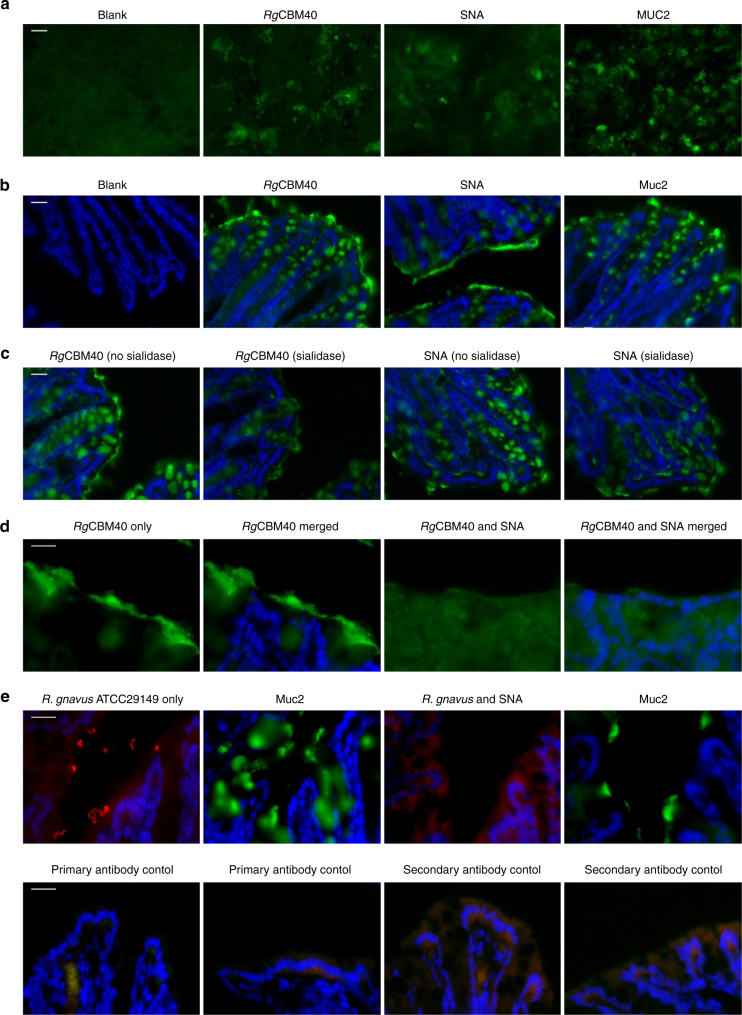
*Rg*CBM40 binding to mucus-producing cells and intestinal tissue sections. **a** Immunostaining pattern for *Rg*CBM40 on LS174T cells correlated with mucin (MUC2) and lectin (SNA) staining, all shown in green. No staining was observed in *Rg*CBM40-free sample (Blank). **b** Immunostaining pattern for *Rg*CBM40 on cryosections of mouse colon correlated with mucin (Muc2) and lectin (SNA) staining, all shown in green. No staining was observed in *Rg*CBM40-free sample (Blank). Cell nuclei were counterstained with DAPI, shown in blue. **c** Sialidase pre-treatment of mouse colonic cryosections markedly reduced the binding of *Rg*CBM40 and SNA lectin. Cell nuclei were counterstained with DAPI, shown in blue. **d** *Rg*CBM40 competition assay with SNA on cryosections of mouse colon. *Rg*CBM40 is shown in green. Cell nuclei were counterstained with DAPI, shown in blue. No *Rg*CBM40 specific staining was detectable when SNA was present. **e** *R. gnavus* binding competition assay with SNA on cryosections of mouse colon. *R. gnavus* ATCC 29149 was incubated on sequential cryosections of mouse colon with or without SNA treatment and is shown in red. The mucus layer is shown in green. Sequential sections were required as both antibodies were raised in the same species. Cell nuclei were counterstained with DAPI, shown in blue. No *R.gnavus* staining was detectable when SNA was present. Appropriate primary antibody and secondary antibody only controls are also shown underneath each panel, showing some background staining. Scale bar: 20 μm

Having shown that *Rg*CBM40 can bind to sialylated oligosaccharides and mucins, we tested its ability to bind to mucus from mouse intestinal tissue and human cell lines cells by immunofluorescence (Fig. 8). Methacarn fixation allowed preservation of mucus in both tissue sections and cell lines. Strong binding was demonstrated to mucus produced by LS174T which correlated with staining patterns of SNA (a sialic acid-specific lectin) and MUC2 (Fig. 8a). No staining was observed in negative controls (*Rg*CBM40 free). *Rg*CBM40, Muc2 and lectin staining was also observed in crypts as well as on the epithelial surface of mouse colonic tissue (Fig. 8b). In addition, sialidase treatment of mouse colonic sections markedly reduced the binding of *Rg*CBM40, as well as the SNA lectin control (Fig. 8c). SNA can outcompete *Rg*CBM40 binding to the mucus layer in mouse colonic tissue sections, further indicating that the binding of *Rg*CBM40 to mucus is sialic acid mediated (Fig. 8d). Similar inhibition was observed when using bacterial cells. *R. gnavus* ATCC 29149 was shown to bind to areas that correlated with mucus staining. This binding was blocked with the addition of SNA (Fig. 8e), confirming the importance of sialic acid recognition in *R. gnavus* ATCC 29149 binding to mucus.

## Discussion

Sialic acids are often found capping mammalian glycans and are thus common binding targets of commensal or invading microbes. A wide variety of microorganisms utilise CBM-containing sialidases to process these terminal sialic acid residues. At present CBMs in family 40 are the only known examples to bind sialic acid and are exclusively associated with sialidases (www.cazy.org). The CBM40 from *R. gnavus*, *Rg*CBM40, adopts the characteristic CBM40 β-sandwich fold, previously reported for CBM40s present in *C. perfringens* ^25,32^, *V. cholerae* ^34^, *M. decora* ^33^, as well as *S. pneumoniae* ^35–37^.

In their description of *C. perfringens Cp*CBM40_NanJ, Boraston et al.^25^ pointed out that there appears to be two subfamilies within the CBM40 family, one typified by *Cp*CBM40_NanJ and the other by *V. cholerae Vc*CBM40_NanH. This was further supported by phylogenetic analyses of all CBM40 structurally characterised so far^32^. It is clear that *Vibrio* sp. forms an outlying clade in the family that has very low amino acid sequence identity (<15%) with the main clade^32^. Here, we showed that the separation between the *Vibrio*-type sequences and canonical CBM40 sequences is also observed across bacterial genomes. Both types adopt a β-sandwich fold, however, this is the most common core fold across CBM families^26^. *Rg*CBM40 crystal structures, of the canonical type, in complex with sialylated ligands, demonstrate shared core binding site residues. In brief, on one side of the sialic acid residue, the carboxylic acid and C4 hydroxyl groups are coordinated by an arginine dyad (Arg128 and Arg204) and a glutamic acid (Glu126) residue, respectively. The importance of the arginine residues was further confirmed by mutational analyses, showing loss of binding of *Rg*CBM40 R204A, *Rg*CBM40 R128A and the double mutant *Rg*CBM40 R128A/R204A to 3′SL. The methyl of the *N*-acetyl moiety and the C-H face of the glycerol moiety reside on a hydrophobic twisted platform surface formed by primarily aromatic residues, of which Tyr116 and Tyr210 are essential for binding. Glu126 was also shown to be essential, as predicted given its conservation and interactions with both the *N*-acetyl group N and the C4 hydroxyl of the sialic acid moiety.

*Rg*CBM40 showed broad specificity for sialylated oligosaccharides with dissociation constants to 3′SL and 6′SL in the millimolar affinity range, 0.57 mM and 1.70 mM, respectively. This is comparable to the affinity recently measured for the isolated *S. pneumoniae Sp*CBM40_NanC^37^ against 3′SL (Kd ~1.5 mM) and 6′SL (Kd ∼1.6 mM). Low sialic acid affinity has also been proposed for *Cp*CBM40_NanJ from the *C. perfringens* sialidase however this was not quantified^25^. Micromolar sialic acid affinity has been observed for *C. perfringens Cp*CBM40_NanI and *S. pneumoniae Sp*CBM40_NanA^32,35^. Additional electrostatic interactions with the sialic acid glycerol moiety may contribute to these unusual affinities, in the case of *Sp*CBM40_NanA via the introduction of a tryptophan in place of *Rg*CBM40 Tyr210 (Supplementary Fig. 3a, b), and in the case of *Cp*CBM40_NanI via Asn158, which approaches the binding site from a nearby loop extension (Supplementary Fig. 3c). *Cp*CBM40_NanI also introduces additional water mediated interactions with the galactose residues of bound 3′SL via a further loop extension (Supplementary Fig. 1h): These are proposed to provide specificity for the corresponding sialic acid linkage^32^. A corresponding extension is absent in *Rg*CBM40 leading to minimal observed interactions between the protein and galactose (Fig.1f, g, Supplementary Fig 1a). Similar absence in *Sp*CBM40_NanA suggests that these water-mediated interactions are not the defining feature of high CBM40 sialic acid affinity.

Overall the binding epitopes of 3′SL and 6′SL, as determined by STD NMR, were in agreement with the crystal structure, and confirmed the flexibility of the galactose and glucose rings at the reducing end. Although the sialic acid moiety was the main recognition element for the interaction with *Rg*CBM40, only weak binding was observed to Neu5Ac or Neu5Gc monosaccharides. Sialic acid residues present in oligosaccharides are α-anomers. However, in solution sialic acid adopts both α- and β-anomeric configurations, as well as an open chain conformation, with the β-anomer forming the dominant constituent^45^. In the *Rg*CBM40 complex crystal structures, sialic acid is bound in the α-anomeric conformation, allowing the axial C2 carboxylic acid moiety to form a conserved interaction with Arg204. The *Rg*CBM40 preference for the minority α-anomer will incur a large entropic penalty. This may provide a major contributory factor to the low observed monosaccharide affinity. Thermodynamic analysis showed that the reaction is driven by enthalpy, with unfavourable entropy (Supplementary Table 2), which is typical of interactions between CBMs and saccharides^46^.

The binding specificity of CBMs most commonly matches that of the appended catalytic module^26,47^. We previously showed that the catalytic activity of *Rg*NanH is specific for α2-3-linked sialic acid^30^. However, our glycan array and STD NMR data clearly showed that *Rg*CBM40 can recognise a wide range of α2-3- and α2-6-sialic acid-linked oligosaccharides which are commonly found in human GI mucins^12,21,23^, suggesting an additional function. More than 100 complex oligosaccharides were identified in mucins from human colonic biopsies where most were mono-, di- or trisialylated^23^. *Rg*CBM40 bound Neu5Acα2-6Tn and Neu5,9Ac_2_α2-6Tn, Neu5Acα2-3TF and Neu5,9Ac_2_-TF9Ac_2_α2-3TF but not to the non-sialylated forms; it also recognises Neu5Ac and acetylated Neu5Ac-linked Lac with α2-3 and α2-6 linkage but shows a strict preference for Neu5Ac-linked LacNAc with α2-3 linkage, in line with the increased expression of group Sd(a)/Cad related epitopes GalNAcα1-4(NeuAcα2-3)Gal along the length of the colon^12^. Despite the large diversity of structures, the sigmoid MUC2 *O*-glycan repertoire and relative amounts in normal individuals is relatively constant^23^, suggesting their role in selecting a specific mucus-associated microbiota. Many bacterial species bind host tissues through protein-carbohydrate interactions via a variety of cell-surface proteins and appendages. Although a wide number of microbial lectins have been functionally and structurally characterised to date, especially from pathogens, only a few carbohydrate-binding proteins present in gut bacteria which interact with mucus have been structurally characterised^13,15^. Interactions between bacterial adhesins from gut commensals and mucin glycans are generally of low affinity, in line with the localisation of these bacteria within the outer mucus layer^48,49^. Here we showed that *Rg*CBM40 could recognise mucins with binding affinity increasing with sialic acid level. Binding was highest towards human colonic MUC2, consistent with the increasing sialic acid gradient along the GI tract from the small intestine to the colon in humans^21^. This study demonstrates CBM40 mediating interaction to mucus, therefore expanding the repertoire of bacterial adhesins to mucus. In addition to variations along the length of the GI tract, mucin sialylation varies significantly between species, and thus could influence host species and niche specificity of the gut symbionts. Interestingly, *Rg*CBM40 also showed binding to Neu5Gc-containing oligosaccharides, albeit to lower affinity as compared to Neu5Ac-oligosaccharides. Humans express predominantly Neu5Ac whereas Neu5Gc is expressed in many non-human mammals^50^. Therefore, the ability of CBM from human gut commensal bacteria to bind to Neu5Gc was unexpected. However, it cannot be excluded that *Rg*CBM40 mediates binding to dietary Neu5Gc-containing glycoproteins^51^.

CBMs typically function to maintain carbohydrate-active enzymes (CAZymes) in proximity of the substrate, thereby enhancing catalytic activity^26,46,52,53^. It has recently been suggested that CBMs may play an additional role in the host-bacterium interaction by not only mediating the attachment of CAZymes to glycans present on host tissues but by aiding the adherence of the entire bacterium^27^. This would be particularly relevant to bacteria of the human gut microbiota which are characterised by their large and diverse repertoires of CBM-containing CAZymes^54^. Many CAZymes are known, or postulated to be, attached to the bacterial cell surface^4^. Here, immunogold labelling confirmed the presence of *Rg*NanH on *R. gnavus* ATCC 29149 cell-surface but not on *R. gnavus* E1. In addition, we showed that the binding of *R. gnavus* ATCC 29149 to intestinal mucus was sialic acid mediated. The potential avidity effect of CBM40-mediated binding of sialylated mucins in vivo (when naturally present on the bacterial cell surface), may favour a mechanism by which CBM40 helps targeting the bacteria towards sialic acid rich regions of the GI tract, therefore promoting bacterial colonisation within the outer mucus layer. Our bioinformatics analyses of bacterial genomes showed that *Rg*CBM40 canonical type domains are widespread among Firmicutes, also reflecting the strong difference in CAZyme content and diversity between the Firmicutes and Bacteroidetes phyla^54^. We thus propose a new role of CBMs in assisting the tropism and spatial distribution of symbiotic bacteria among physical niches in the gut.

## Methods

### Materials

General chemicals including Neu5Ac were from Sigma (St Louis/MOI, US). Neu5Gcα2-3Lac Neu5Gcα2-6Lac, Neu5Ac-STn, Neu5Gc-STn and STFαOC3H6N3) were synthesised following published methodology^38,55^. Neu5Gc, 3′SL, 6′SL, 3′SGal, 6′SGal, 3′SLN, 6′SLN, were from Carbosynth (Compton, Berkshire, UK). 2,7-anhydro-Neu5Ac was synthesised as previously reported^31^. Sialidase from *Clostridium perfringens* and *Salmonella typhimurium* LT2 were from New England Biolabs (Ipswich, MA, USA). Sialidase 0625 from *Akkermansia muciniphila* was a gift from WM de Vos^30^. Polyclonal antiserum against IMAC-purified His_6_-*Rg*NanH^30^ was raised in rabbits by BioGenes GmbH (Berlin, Germany) and provided at a titre of >1:200,000. Protease inhibitors benzamidine, *N*-ethylmaleimide, PMSF, sodium azide and soy bean inhibitor were from Sigma. Fluorescein labelled *Sambucus nigra* lectin (SNA-FITC) biotinylated SNA (SNA-biotin) and Vectashield were from Vector laboratories (Peterborough, UK). Streptavidin Alexa Fluor 488 conjugate was Thermo Fischer Scientific (Eugene/OR, USA). Deuterium oxide (99.9% 2H) and Tris(hydroxymethyl-d3)amino-d2-methane (Tris-d11, 98% 2H) were from Sigma. Mouse monoclonal anti-His-HiLyte Flour 555 antibody was obtained from LifeSpan BioSciences (Seattle/WA, USA). Anti-rabbit IgG Alkaline Phosphatase (AP) conjugate antibody was from Sigma. Blocking reagent was from Perkin Elmer (Boston/MA, USA). Rabbit Mucin 2 antibody H-300 was from Santa Cruz (Dallas/TX, US, SC-15334), goat anti-rabbit IgG secondary antibody, Alexa Fluor 488 (A11034) and goat anti-rabbit IgG secondary antibody Alexa Fluor 594 (A11037) from Thermo Fischer Scientific. DAPI was from Life Technologies, O.C.T. Compound from VWR and Hydromount from National Diagnostics (Atlanta/GA, USA).

### Expression and purification of *Rg*CBM40 and *Rg*NanH

Using the full-length sequence encoding *Rg*NanH in pOPINF from *R. gnavus* strain ATCC 29149 as a template^30^, *Rg*CBM40 (residues 50–237), *Rg*NanH (residues 26–723) and *Rg*GH33 (residues 243–723) were cloned into the pEHISTEV vector^56^ using the primers listed in Supplementary Table 4. Protein expression and purification of *Rg*CBM40 and *Rg*NanH was similar to that of *Rg*GH33^30^. Points of divergence are indicated below. For protein expression, recombinant plasmids were transformed into *E. coli* BL21 Rosetta (DE3) (Novagen, NJ, USA). A single colony was used to inoculate a 10 ml Luria Bertani (LB) medium pre-culture, which was incubated overnight under shaking at 200 r.p.m. (at 30 °C for crystallisation and protein size determination or at 37 °C for all other protein assays). The pre-culture was used to inoculate 500 ml of auto induction medium (Formedium, Norfolk, UK), which was incubated under shaking at 37 °C for 3 h followed by 60 h incubation at 16 °C. All cultures were inoculated with 50 µg ml^−1^ kanamycin.

For crystallisation and protein size determination, cells were collected by centrifugation, resuspended in phosphate buffered saline (PBS, 150 mM sodium chloride, 10 mM sodium phosphate, pH 7.4) for *Rg*CBM40 and in 20 mM Tris-HCl pH 7.5, 50 mM NaCl for *Rg*NanH, supplemented with DNase I (20 µg ml^−1^) and cOmplete protease inhibitor mixture tablets (Roche, Welwyn Garden City, UK), and lysed using a constant flow cell disrupter. Insoluble components were removed by centrifugation and filtration through a 0.22 µm pore size syringe driven filter (Millipore, NJ, USA). Soluble lysate was loaded onto a nickel-Sepharose column (GE Healthcare, Little Chalfont, UK) overnight at 4 °C. The sample was then washed extensively with lysis buffer supplemented with 5 mM imidazole for *Rg*CBM40 and with 150 mM imidazole for *Rg*NanH and was eluted using lysis buffer supplemented with 50 mM imidazole for *Rg*CBM40 and with 300 mM imidazole for *Rg*NanH. The sample was then dialysed into lysis buffer and cleaved of its six-histidine tag using in-house Tobacco Etch Protease at a mass ratio of 1:50 overnight at 4 °C. Finally, the gel filtration step using a Sephacryl S-100 column (GE Healthcare) was performed using 20 Tris, pH 7.5 with 50 mM NaCl. The purified *Rg*CBM40 was crystallised as described below. To determine the size in solution of *Rg*NanH, size exclusion chromatography with multi angle light scattering (SEC-MALS) was performed using an NGC chromatography system (Biorad, Hercules, CA, USA) equipped with a DAWN HELEOS II MALS detector (Wyatt technology, Haverhill, UK) and an Optilab T-rEX differential Refractive Index detector (Wyatt Technology). The data were analysed using ASTRA (Wyatt Technology).

For all other protein assays, the cell pellets were resuspended in Bug buster-HT (Merck, Kenilworth, NJ, USA) with the supplied lysozyme and lysed by shaking in this solution for 1 h at room temperature. Insoluble material was removed by centrifugation at 4 °C, 3320×*g* for 25 min and the supernatant was dialysed into desalting buffer (50 mM Tris-HCl, 150 mM NaCl, pH 7.8 containing 10 mM imidazole for *Rg*GH33 and *Rg*NanH and no imidazole for *Rg*CBM40, the difference is due to the poor binding of the His_6_-tag of *Rg*CBM40 to the nickel column) to remove the Bug buster-HT. Again insoluble material was removed by centrifugation as above, except at 8000×*g*. Purification of the soluble lysate was loaded onto the immobilised metal ion affinity chromatography (IMAC column, His-bind, Novagen) in binding buffer (desalting buffer with the addition of 10 mM imidazole) using the Akta Express (GE Healthcare). The protein was eluted with binding buffer containing 500 mM imidazole and then immediately desalted into desalting buffer. The partially purified protein was concentrated using 3.5 kDa MWCO spin columns (Sartorius, Gottingen, Germany) prior to gel filtration again with the Akta Express in desalting buffer (see above) on a Superdex 75 column (GE Healthcare). Purity of the proteins was assessed throughout by SDS-PAGE using the Novex system (Thermo Fisher Scientific).

### Site-directed mutagenesis

Site directed mutagenesis of *Rg*GH33 to introduce the D282A mutation in the active site was carried out using the QuikChange kit, following the manufacturer’s instructions, Agilent (Santa Clara, CA, USA). Site-directed mutants of *Rg*CBM40; I95A, Y116A, E126A, R128A, R204A and double mutant R128A/R204A, were obtained from NZyTech (Lisbon, Portugal). The primers are listed in Supplementary Table 4. The integrity of the *Rg*GH33 and *Rg*CBM40 mutants was checked by circular dichroism (CD).

### Circular dichroism

CD spectra were recorded using a JASCo J-700 spectropolarimeter, under the following conditions: 20 nm/min scan speed, bandwidth 1 nm, response 2 s, 5 points/nm and 4 accumulations. Far-UV spectra (260-180 nm) were recorded in a 0.1 mm pathlength cell. The spectropolarimeter was calibrated using camphorsulphonic acid (Sigma). The protein was extensively dialysed into 10 mM sodium phosphate buffer, pH 6.5 and a buffer only control was subtracted from all spectra using the molar CD factor calculated as follows: (113 × 30 × 10^−6^)/ [conc(mg ml^−1^)×pathlength (cm)].

### Protein crystallisation

The final crystallisation condition was 0.2 M ammonium chloride with 20% PEG 8000. The drop contained 0.5 µl protein solution at 25 mg ml^−1^ and 0.5 µl reservoir solution, initial crystals grew in 4 weeks and growth time was improved significantly using micro seeding^57^. Crystals were cryoprotected using the crystallisation condition supplemented with 25% (w/w) glycerol. To achieve crystal structures in complex with 3′SL and 6′SL, the crystals were grown in crystallisation condition supplemented with 20 mM ligand followed by a 60 min soak in crystallisation condition supplemented with 100 mM ligand immediately prior to cryoprotection and mounting.

### Solving the crystal structure

X-ray diffraction experiments were performed at 100 K. Data were collected using a Rigaku MSC Micromax 007 HF X-ray source, with a fixed wavelength of 1.542 Å, and a Saturn 944+ CCD detector. Sweeps were indexed and integrated separately and then scaled together within the HKL2000 data processing package^58^. Phasing was performed by Phaser^59^ within the CCP4 package^60^ using the CBM40 of the *M. decora* sialidase NanL (*Md*CBM40_NanL) (PDB 2SLI)^33^ as the molecular replacement model. The model was refined using iterative cycles of Refmac5^61^ and Coot^62^. The PDB REDO server was used to optimize the refinement parameters^63^. The model was validated using the Molprobity server^64^. Paired refinement performed by the PDB REDO server indicated that the models were improved by the inclusion of high resolution, low completeness data for the 3′SL and 6′SL complexes^65^. For an illustrative stereo image of a portion of the electron density map, see Supplementary Fig. 13.

### Isothermal titration calorimetry

Isothermal titration calorimetry (ITC) experiments were performed using the PEAQ-ITC system (Malvern, Malvern, UK) with a cell volume of 200 µl. Prior to titration protein samples were exhaustively dialysed into PBS. The ligand was dissolved in the dialysis buffer. The cell protein concentration was 115 µM (except for mutant I95A where it was 173 µM and the wild-type interaction with 6′SL where it was 230 µM) and the syringe ligand concentration was 10 mM (25 mM for Neu5Ac). Controls with titrant (sugar) injected into buffer only were subtracted from the data. Analysis was performed using Malvern software, using a single-binding site model. The stoichiometry of binding sites was set to 1.0 as this was evident from the crystal structure. Quantitative and most qualitative experiments were carried out in triplicate.

### STD NMR experiments

^1^H and ^13^C resonance assignment for all the sugars was performed on the bases of 1D ^1^H, 2D DQF-COSY, TOCSY, HSQC and NOESY experiments run on the free ligands in unbuffered D_2_O, pH 7.0. For STD NMR experiments, all the samples consisted of 1 mM sialoglycans and 50 μM *Rg*CBM40 (WT or I95A mutant) in D_2_O buffer solution of 10 mM Tris-d_11_ pH 7.8 and 100 mM NaCl (ligand: protein ratio 20:1). An STD pulse sequence that included 2.5 ms and 5 ms trim pulses and a 3 ms spoil gradient was used. Saturation was achieved applying a train of 50 ms Gaussian pulses (0.40 mW) on the f2 channel, at 0.60 p.p.m. (on-resonance experiments) and 40 p.p.m. (off-resonance experiments). The broad protein signals were removed using a 40 ms spinlock (T1ρ) filter. All the experiments were recorded at ^1^H frequency of 800.23 MHz on a Bruker Avance III spectrometer equipped with a 5 mm probe TXI 800 MHz H-C/N-D-05 Z BTO, at 288 K. For all the sialoglycans in the presence of *Rg*CBM40, an STD experiment with a saturation time of 2 s and a relaxation delay of 5 s was performed, as a first test for binding. For the confirmed binders, the STD NMR experiments were carried out at different saturation times (0.5, 1, 2, 3, 4 and 5 s) with 1 K scans and relaxation delay of 5 s, in order to obtain the binding epitope mapping. The resulting build-up curves for each proton were fitted mathematically to a mono-exponential equation (*y* = *a*×[1-exp(*b*×*x*)]), from which the initial slopes (*a*×*b*) were obtained. For each ligand, the binding epitope mapping was obtained by dividing the initial slopes by the one of the H7 proton of the corresponding sialic acid ring, to which an arbitrary value of 100% was assigned. This normalisation of the STD values allows the comparison across all the sialoglycans.

### Structure-based sequence alignment and bioinformatics analyses

A structural alignment of *Rg*CBM40 was carried out with all CBM40 structures available to date (Results and Supplementary Methods). This served as a basis for producing an alignment including both canonical and *Vibrio*-type CBM40 sequences to create a profile Hidden Markov Model (pHMM) using the HMMER3 software (http://hmmer.org/) (Supplementary Fig. 14), intended to detect both types simultaneously and ensure that hit sequences of both types are thus properly aligned for subsequent comparative analysis. Additionally, we created pHMMs corresponding to the canonical-only and *Vibrio*type-only CBM40 sequences of this alignment, to resolve the type of each hit. Protein domain databases such as Pfam^66^ currently characterise the canonical CBM40 as a sequence family belonging to a larger superfamily (“clan”), and some individual domains make good matches to more than one related family, i.e., including non-CBM40 such as “Concanavalin A-like lectin/glucanases” (in contrast, no Pfam domain clearly defines the *Vibrio* CBM40). We therefore also used the corresponding Pfam pHMMs, as well as our own, to search all available (177 million) protein sequences from annotated NCBI prokaryote genomes, using HMMER3. Where individual hit domains matched multiple pHMMs, we compared scores to identify and discard hits which might be better regarded as related, non-CBM40 domains. The remaining CBM40 proteins were screened for the presence of the sialidase domain (GH33), as previously described^30^. We reduced this to a nonredundant set (Supplementary Methods) for further analysis. A detailed phylogenetic analysis is beyond the scope of this study, but we estimated evolutionary distances between these 51 representative sequences using fprotdist in EMBASSY-PHYLIP^67,68^ from which the tree was calculated by neighbour-joining (fneighbor). All sites were included in the analysis, using the PMB model with a uniform rate of evolution. This was repeated on 1000 replicate datasets produced by bootstrap resampling (fseqboot; consensus tree produced by fconsense). The figure was produced with FigTree (http://tree.bio.ed.ac.uk/software/figtree/). Bioinformatics analyses were performed using the Gut Health and Food Safety Linux servers at Quadram Institute Bioscience.

### Glycan microarray screening

Glycan microarrays were fabricated using epoxide-derivatized slides as previously described^38^. Printed glycan microarray slides were blocked by ethanolamine, washed and dried. Slides were then fitted in a multi-well microarray hybridisation cassette (AHC4X8S, ArrayIt, Sunnyvale, CA, USA) to divide into 8 subarrays. The subarrays were blocked with ovalbumin (1% w/v) in PBS (pH 7.4) for 1 h at room temperature, with gentle shaking. Subsequently, the blocking solution was removed and diluted protein samples of *Rg*CBM40 and *Rg*GH33 D282A with various concentrations were added to each subarray. After incubating the samples for 2 h at room temperature with gentle shaking, the slides were washed. Diluted anti-His-HiLyte Flour 555 antibodies in PBS were added to the subarrays, incubated for 1 h at room temperature, washed and dried. The microarray slides were scanned by Genepix 4000B microarray scanner (Molecular Devices Corp., Union City, CA, USA). The data analysis was performed using Genepix Pro 7.0 analysis software (Molecular Devices Corp.). It is important to note that glycans on the array with sialic acid *O*-acetyl groups undergo gradual losses of these labile ester groups. Therefore, definitive conclusions about 9-O-acetylation are only possible in instances wherein binding is exclusively to the O-acetylated sialoglycan spot, and not to the corresponding non-O-acetylated spot.

### *Rg*CBM40 binding to mucus-producing cells

The binding of *Rg*CBM40 to mucus-producing LS174T cell line (80% confluent, passage 12) was performed by incubating the cells with 150 µg ml^−1^ *Rg*CBM40 in cell culture medium for 2 h at 37 °C. Control samples were incubated with cell culture medium only. The cells were then washed with PBS, fixed in methacarn (60% dry methanol, 30% chloroform and 10% acetic acid) and washed in PBS containing 0.05% bovine serum albumin (BSA). Blocking was done with TNB buffer (0.5% w/v blocking reagent in 100 mM Tris-HCl, pH 7.5, 150 mM NaCl) supplemented with 5% goat serum. The *Rg*CBM40 binding was detected with custom-made rabbit *Rg*NanH antiserum diluted 1:100 in PBS and goat anti-rabbit antibody diluted 1:400 in PBS. The same antibodies were used for negative control sample (*Rg*CBM40-free). In the lectin control sample, SNA-biotin (incubated at 75 µg ml^−1^) was detected with streptavidin conjugate (2.5 µg ml^−1^). MUC2 was detected with rabbit Mucin 2 antibody diluted 1:50 in PBS and goat anti-rabbit antibody diluted 1:200 in PBS. The cells were counterstained with DAPI and mounted in Vectashield. The slides were imaged using a Zeiss Axio Imager 2 microscope.

### *Rg*CBM40 and *R. gnavus* binding to intestinal tissue

To assess the binding of *Rg*CBM40 to intestinal tissue sections, colon of wild-type C57BL/6 mouse was washed with PBS, fixed in methacarn, embedded in O.C.T. compound and cut into 8 µm sections. Access to mouse tissues was carried out under the Animal Welfare and Ethical Review Body of University of East Anglia's establishment licence (according to Home Office requirements). Tissue sections were washed in PBS containing 0.05% BSA and blocked with TNB buffer (0.5% w/v blocking reagent in 100 mM Tris-HCl, pH 7.5, 150 mM NaCl) supplemented with 5% goat serum. The slides were then washed in PBS 0.05% BSA, followed by 2 h incubation of 150 µg ml^−1^ *Rg*CBM40 in PBS at 37 °C. Control tissue sections were incubated in PBS only. After washes in PBS with 0.05% BSA, the binding of *Rg*CBM40 was detected with custom-made rabbit *Rg*NanH antiserum (diluted 1:100 in TNB buffer) and goat anti-rabbit antibodies (diluted 1:200 in PBS). Negative control sample (*Rg*CBM40-free) was also incubated with these primary and secondary antibodies. Muc2 was detected with Mucin 2 antibody diluted 1:100 in TNB buffer and goat anti rabbit antibody diluted 1:200 in PBS. In lectin controls SNA-FITC was incubated at 4 µg ml^−1^. The sections were counterstained with DAPI and mounted in Hydromount mounting medium. The slides were imaged using an Axio Imager 2 Zeiss microscope. To assess the binding specificity of *Rg*CBM40 to sialylated structures, the tissue sections were pre-treated with sialidase. Briefly, saponification was performed to make the enzymatic digestion of mouse colonic tissue sections effective^69^. The sections were treated with 0.5% KOH in 70% ethanol for 15 min at room temperature. After three PBS washes, 500 U ml^−1^ sialidase from *Clostridium perfringens* in GlycoBuffer 1 (New England Biolabs) was added and incubated for 14 h at 37 °C. Sections were incubated in sialidase-free GlycoBuffer 1 under the same experimental conditions and used as a control of sialidase digestion to assess the binding of *Rg*CBM40 and SNA to tissue sections as described above.

To assess the binding of *R. gnavus* to intestinal tissue sections, colon of wild-type C57BL/6 mouse was washed with PBS, fixed in methacarn, embedded in O.C.T. compound and cut into 12 µm sections. Tissue sections were washed in PBS, then incubated with SNA in PBS at 20 μg ml^−1^ for 1 h. Prior to incubation with bacteria, the slides were washed with PBS. *R. gnavus* ATCC 29149 was cultured anaerobically in BHI-YH media for 24 h as previously described^29^. The culture was then then used to inoculate YCFA media supplemented with 3′SL at a concentration of 7 mg ml^−1^, and cultured for 20 h. The bacteria were then washed twice with fresh YCFA, and resuspended at an OD of 1. The tissue sections were then transferred in a humid chamber to the anaerobic cabinet, and the bacteria incubated on the sections for 1 h at 37 °C. The slides were then washed twice with YCFA and fixed with 4% paraformaldehyde in PBS for 15 min. The slides were transferred out of the anaerobic cabinet, then washed with PBS and blocked with TNB buffer (0.5% w/v blocking reagent in 100 mM Tris-HCl, pH 7.5, 150 mM NaCl) supplemented with 5% goat serum. The presence of *R. gnavus* and Muc2 was detected with custom-made rabbit *Rg*NanH antiserum (diluted 1:100) and Mucin 2 antibody (1:100), respectively. Goat anti-rabbit antibodies (diluted 1:500) were used for immunodetection. The sections were counterstained with DAPI and mounted in Prolong gold anti-fade mounting medium. The slides were imaged using an Axio Imager 2 Zeiss microscope, using a ×63 objective.

### Mucin purification

Culture media from LS174T cell line were freeze-dried before extraction of MUC2. After freeze-drying, samples were solubilised overnight in 6 M guanidine chloride (GuCl) buffer containing protease inhibitors (7.95 mM EDTA, 12.25 mM benzamidine, 6.25 mM *N*-ethylmaleimide, 1.25 mM PMSF, 3.75 mM sodium azide, 0.1 mg ml^−1^ soy bean inhibitor). Samples were centrifuged at 18 500×*g*. The pellet was reduced with dithiothreitol (DTT) at 10 mM for 4 h at 45 °C and alkylated with 25 mM iodoacetamide overnight before dialysis against 50 mM ammonium bicarbonate. The same protocol was followed for purifying mucins from the scraped mucus from small intestine and colon of mouse models. The supernatants containing soluble mucins were diluted in 4 M guanidinium chloride (GuCl) with phosphate buffered saline (PBS) and adjusted with cesium chloride at 1.4 g ml^−1^ density. Supernatants were subjected to an ultracentrifugation (Beckman, Brea, US) at 234,000×*g* for 72 h at 20 °C. Fractions of 1 ml were collected and weighed. Fractions between 1.35 and 1.45 g ml^−1^ were kept and dialysed against 50 mM ammonium bicarbonate. These fractions contained the purified mucins.

### Release of oligosaccharides from mucin

The mucins were subjected to β-elimination under reductive conditions (0.1 M sodium hydroxide, 1 M sodium borohydride) for 20 h at 45 °C. The reaction was stopped by adding Dowex 50 × 8 (Sigma) and filtered before being co-evaporated with methanol 3 times. Remaining salts were removed by Carbograph (Grace, Columbia, USA).

### Permethylation of *O*-glycans

Permethylation was performed on released *O*-glycans from the different mucins samples. Samples were solubilized in 200 μl dimethyl sulfoxide. Then sodium hydroxide (trace of powder) and 300 μl iodomethane were added in anhydrous conditions and the samples vigorously shaken at room temperature for 90 min. The permethylation reaction was stopped by addition of 1 ml acetic acid (5% vol/vol). Permethylated *O*-glycans were purified on a Hydrophilic-Lipophilic Balanced (HLB) Oasis cartridge (Waters, Milford, USA). Briefly, cartridges were activated by methanol, equilibrated with methanol:water (5:95, vol:vol), and samples loaded onto the cartridges. Cartridges were washed by methanol:water (5:95, vol:vol) and the permethylated *O*-glycans eluted by methanol.

### Analysis of permethylated *O*-glycans by mass spectrometry

MALDI-TOF and TOF/TOF-MS data were acquired using the Bruker Autoflex analyzer mass spectrometer (Applied Biosystems, Foster City, CA, USA) in the positive-ion and reflectron mode by using 2,5-dihydroxibenzoic acid (DHB; Sigma; 10 mg ml^−1^ in 70:30 methanol:water) as the matrix. The relative quantification of sialylation on mucins was calculated based on the sum of all areas of mass peaks corresponding to sialylated structures divided by the sum of all areas of mass peaks corresponding to defined *O*-glycans.

### Enzyme linked immunosorbent assay

*Rg*CBM40 binding to purified mucins was tested by ELISA. Mucins (100 μl of 10 μg ml^−1^) were immobilised onto a high binding 96 well plate (Greiner, Stonehouse, UK) overnight at 4 °C. All subsequent steps were carried out for 1 h at room temperature. The plates were blocked with 3% (w/v) BSA, incubated with *Rg*CBM40 (500 μg ml^−1^), followed by an incubation with 1:5000 anti-*Rg*NanH (raised in rabbit, Biogenes) then with 1:5000 anti-rabbit secondary antibody (raised in donkey) conjugated to peroxidase (GE Healthcare). Between each step, the plate was washed with 3 × 300 μl of PBS containing 0.05% (v/v) Tween 20 (PBST). Prior to detection, an additional wash step and 30 s incubation with PBST was carried out. Binding was detected using tetramethylbenzidine (TMB) visualisation solution (Biolegend, San Diego, CA, USA) which was incubated for 15 min. The reaction was stopped by addition of 2 M H_2_SO_4_ and absorbance measured at 450 nm using a plate-reader (Bench Marl Plus, Biorad), subtracting background readings at 570 nm. Negative controls including no *Rg*CBM40 (subtracted from A_450_ value), no primary or no secondary antibody were carried out in parallel. For comparison between plates, values were normalised to the reading for LS174T MUC2 which was arbitrarily set at 100%. For enzymatic treatment of the mucin, LS174T MUC2 (2 mg ml^−1^) was incubated with sialidases (2 µg ml^−1^) overnight at 4 °C on a rotary wheel prior to immobilisation on the plate. For chemical treatment of mucin, LS174T MUC2 was incubated with 0.1 M trifluoroacetic acid (TFA) at 80 °C for 1 h, dialysed against ammonium bicarbonate (50 mM), lyophilised and redissolved in H_2_O. For the competition assays, *Rg*CBM40 was incubated with 1 mM of free sugar overnight at 4 °C on a rotary wheel prior to addition to the ELISA plate as above. Experiments were carried out in triplicate.

### HPAEC-PAD analyses

The substrates, 3′SL (500 μM, 8.5 nM enzyme), 3′SLX (Neu5Ac form), (500 μM, 80 nM enzyme) or LS174T MUC2 (0.9 mg ml^−1^, 1.5 nM enzyme) were incubated with *Rg*NanH or *Rg*GH33 at 37 °C in 20 mM sodium phosphate buffer, pH 6.5. BSA (0.1 mg ml^−1^) was included in the oligosaccharide reactions. Control reactions without enzyme were also carried out in parallel. Aliquots of reaction were removed and the reaction terminated by boiling for 20 min. For LS174T MUC2, the released sugars were removed using 5 kDa MWCO spin columns and the remaining mucin subjected to acid hydrolysis; the samples were incubated with 0.1 M HCl at 80 °C for 1 h, dried under vacuum and resuspended in H_2_O at 1 mg ml^−1^. The amount of Neu5Ac remaining on the mucin was quantified by comparing the peak size for Neu5Ac with an internal standard of 2-keto-3-deoxynononic acid (Kdn). The reaction products for all substrates were filtered with 0.22 μm spin tubes prior to analysis by HPAEC-PAD (Dionex ICS-5000, Thermo Fisher Scientific). An internal standard of fucose (50 μM) was used for 3′SL and 3′SLX. For 3′SL, a Carbo-Pac PA1 column (Thermo Fisher Scientific) was used with a 6 min isocratic gradient of 100 mM sodium hydroxide, 100 mM sodium acetate followed by a 10 min washing step with 100 mM sodium hydroxide, 200 mM sodium acetate and 10 min re-equilibration with 100 mM sodium hydroxide, 100 mM sodium acetate. For 3′SLX, a Carbo-Pac PA100 was used with 5 min at 100 mM sodium hydroxide, a gradient of 0–50 mM sodium acetate over 5 min, followed by a gradient of 50–225 mM sodium acetate. The column was then cleaned with 500 mM sodium acetate for 5 min and re-equilibrated for 15 min at 100 mM sodium hydroxide. For analysis of the acid hydrolysis products of MUC2, a Carbo-Pac PA10 was used with a gradient of 70–300 mM sodium acetate with 100 mM sodium hydroxide over 10 min, a brief (1 min) period of 300 mM sodium acetate followed by a decrease (over 1 min) to 70 mM sodium acetate and 15 min re-equilibration at 70 mM sodium acetate. All columns were protected with their respective guard columns, except for the mucin analysis where an amino-guard column was used.

### Western blotting

*R. gnavus* strains were grown to stationary phase and cells pelleted by centrifugation for 10 min at 3000×*g* at 4 °C. The supernatant was collected and the extracellular proteins concentrated 50-fold using a 10-kDa MWCO Amicon Ultra-0.5 Centrifugal Filter (Millipore, Watford, UK). The cell pellet was resuspended in 20 μl PBS with an equal bead (100 μm glass beads) volume added and samples vortexed at full speed three times for 2 min with 2 min rest intervals on ice. The volume was made up to 17 μl per mg wet cell weight with PBS and vortexed at full speed again for 2 min. The beads were removed by allowing them to settle under gravity and the remaining samples centrifuged for 30 min at 17,000×*g* at 4 °C. The supernatant containing the soluble cytosolic proteins was collected and concentrated 10-fold using a 10-kDa MWCO Amicon Ultra-0.5 Centrifugal Filter. The remaining pellet was dissolved in 1.7 μl per mg wet cell weight digestion buffer (50 mM Tris-HCl, (pH 8.0), 5 mM MgCl_2_, 5 mM CaCl_2_, 10 mg ml^−1^ hen egg white lysozyme (Sigma)), and incubated at 37 °C for 3 h. The samples were centrifuged for 30 min at 17,000×*g* at 4 °C, and the supernatant containing the cell wall associated proteins collected. Samples were analysed on duplicate on NuPAGE Novex 4–12% Bis-Tris gels, one gel was stained with InstantBlue stain (Expedeon, Swavesey, UK) and the other gel blotted onto a PVDF membrane using X-cell II Blot module (Thermo Fisher Scientific), according to manufacturer’s instructions. Membranes were blocked with 3% BSA in PBST for 3 h, and then incubated with the custom-made anti-*Rg*NanH antibody raised in rabbit diluted 1:5000 in 1% BSA in PBST overnight. Blots were washed in PBST, then incubated with anti-rabbit IgG AP-conjugate antibody (Sigma) diluted 1:7500 in 1% BSA in PBST for 2 h. After washing three times in PBST, the blots were incubated using a visualisation solution (10 ml of 0.1 M Tris-HCl (pH 9.6), 40 μl of 1 M MgCl_2_, 20 μl of nitroblue tetrazolium, and 10 μl of 5-Bromo-4-Chloro-3-Indolyl phosphate, Sigma) for up to 15 min, and washed in distilled water to stop the development of the signal.

### Immunogold labelling of whole bacterial cells

*R. gnavus* strains were grown to stationary phase and cells pelleted by centrifugation for 10 min at 3000×*g* at 4 °C before being resuspended in PBS. A small drop of concentrated *R. gnavus* cell suspension was applied to a formvar/carbon coated gold TEM grid (Agar Scientific, Stansted, UK) and left for 1 min. The bacteria on the grids were vapour fixed by placing the grids in a sealed Petri dish with a small cap-full of 25% glutaraldehyde (Agar Scientific) for 2 h. The grids were floated on drops of 50 mM Glycine/PBS for 15 min followed by floating on drops of Aurion blocking buffer (Aurion, Wageningen, The Netherlands) for 30 min. The grids were then washed five times for 5 min with 0.1% BSA-C (Aurion) in PBS. Grids were incubated in anti-*Rg*NanH antibody raised in rabbit diluted 1:2000 with 0.1% BSA-C/PBS or in a control solution of 0.1% BSA-C/PBS overnight at 4 °C. The grids were washed five times for 5 min with 0.1% BSA-C/PBS. Grids were then transferred to a 1/50 dilution of goat anti-rabbit antibody conjugated with 10 nm gold balls (Agar Scientific) in 0.1% BSA-C/PBS and incubated for 2 h at room temperature. The grids were washed five times for 5 min with 0.1% BSA-C/PBS, followed by three 5 min washes in PBS only. The grids were refixed by immersing them in 2% glutaraldehyde/PBS for 1.5 h followed by three 5 min PBS washes and three 5 min distilled water washes before the grids were carefully blotted and dried. The grids were examined and imaged in a FEI Tecnai G2 20 Twin transmission electron microscope at 200 kV.

### Statistical analysis

One-way ANOVA model analyses were used to assess the binding of *Rg*CBM40 to purified mucins by ELISA. When the effect of the factor was found to be significant (*p* value < 0.05) and its number of levels greater than 2, a Tukey test was used to assess the significance of the difference between multiple means. Statistical analyses were performed using the software SAS 9.4 (NC, USA).

### Data availability

Atomic coordinates have been deposited in the Protein Data Bank (www.rcsb.org) with accession codes: 6ER2 (unbound), 6ER3 (3′SL bound) and 6ER4 (6′SL bound). The data that support the findings of this study are available from the corresponding author upon request.

## Electronic supplementary material

Supplementary Information
