# Supplementary material for: Unravelling the specificity and mechanism of sialic acid recognition by the gut symbiont Ruminococcus gnavus
Source: Nat Commun. 2017 Dec 19;8:2196. doi: 10.1038/s41467-017-02109-8 (PMC5736709; doi:10.1038/s41467-017-02109-8)
Supplement: Supplementary file 1 — Supplementary Information [file 41467_2017_2109_MOESM1_ESM.pdf]

Supplementary Information

Supplementary Figures

Supplementary Figure 1 Comparison of CBM40 crystal structures indicating conservation of the β-sandwich fold

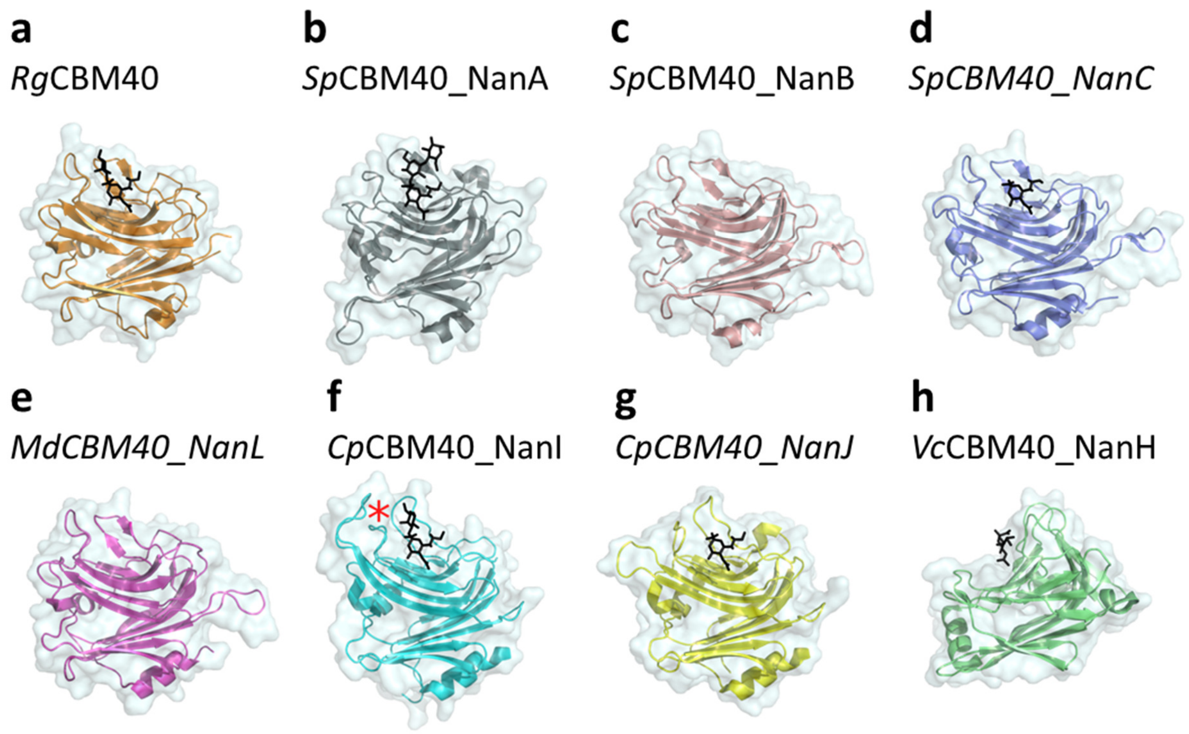

(**a**) *Rg*NanH_CBM40 (*Rg*CBM40) with 3'SL, (**b**) *Sp*CBM40_NanA with 3'SL, (**c**) *Sp*CBM40_NanB, (**d**) *Sp*CBM40_NanC with Neu5Ac, (**e**) *Md*CBM40_NanL, (**f**) *Cp*CBM40_NanI with 3'SL, (**g**) *Cp*CBM40_NanJ with Neu5Ac, and (**h**) *Vc*CBM40_NanH with Neu5Ac. Ligands are represented by black sticks. For the *Cp*CBM40_NanI image the loop indicated to form additional water mediated hydrogen bonding interactions with the sialyllactose galactose residue is indicated with a star.

Supplementary Figure 2 Comparison of CBM40 sialic acid binding sites

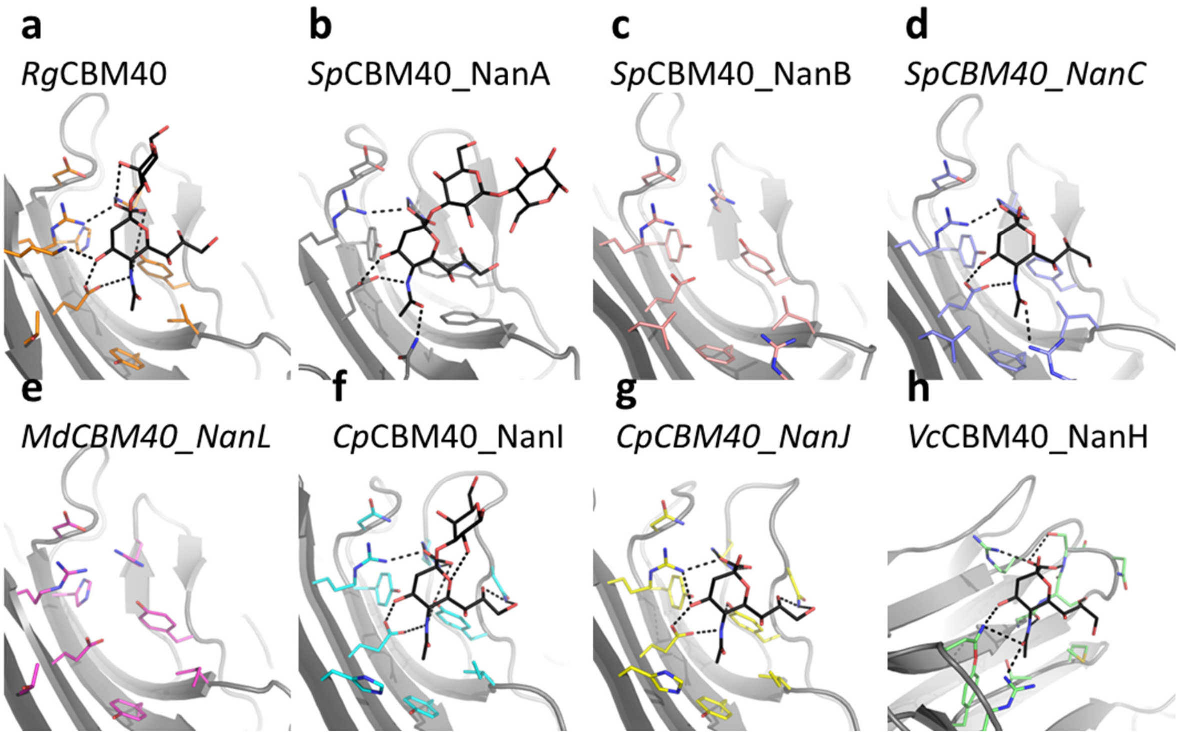

(**a**) *Rg*CBM40 with 3'SL, (**b**) *Sp*CBM40_NanA with 3'SL, (**c**) *Sp*CBM40_NanB, (**d**) *Sp*CBM40_NanC with Neu5Ac, (**e**) *Md*CBM40_NanL, (**f**) *Cp*CBM40_NanI with 3'SL, (**g**) *Cp*CBM40_NanJ with Neu5Ac, and (**h**) *Vc*CBM40_NanH with Neu5Ac. Ligands are represented as black sticks with hydrogen bonding interactions as black dashed lines.

Supplementary Figure 3 Comparison of the Neu5Ac glycerol environment of the ligand in complex with CBM40 structures

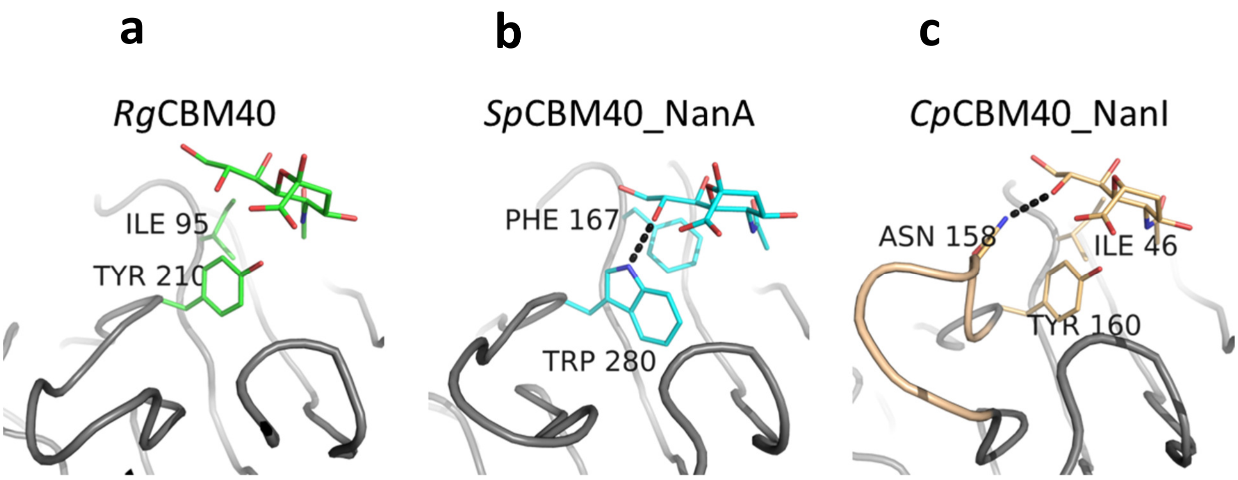

(**a**) *Rg*CBM40 (**b**) *Sp*CBM40_NanA, and (**c**) *Cp*CBM40_NanI. Ligands and interacting residues are coloured. Dashed black lines indicate hydrogen bonding interactions.

Supplementary Figure 4. Alignment of a subset of the domain hits of the combined-CBM40 pHMM

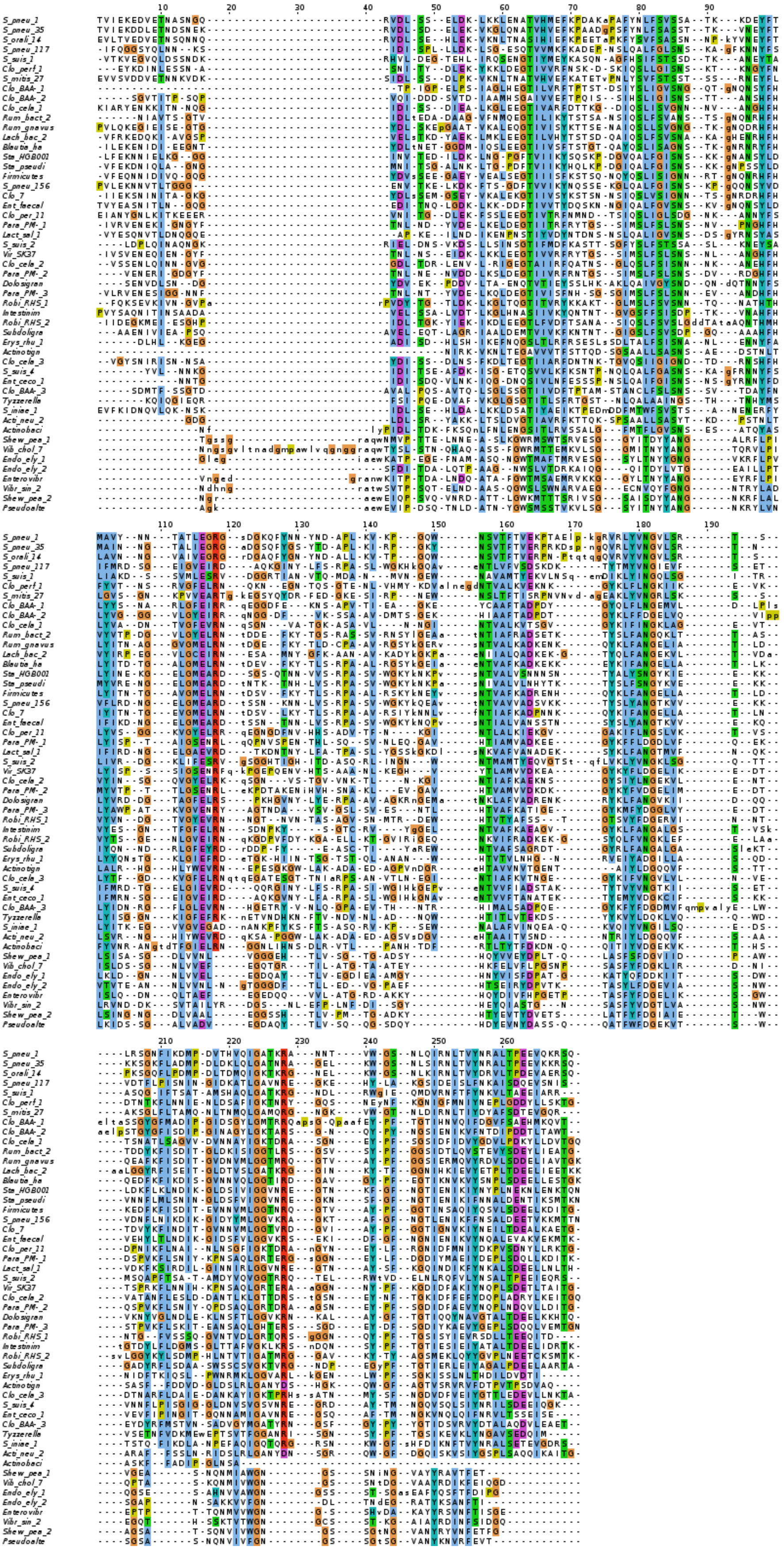

This alignment consists of 51 nonredundant domain sequences from the complete alignment of all domain hits produced by hmmsearch of HMMER3, using the combined-model (canonical and Vibrio-type CBM40) as the query. Refer to **Supplementary Methods** for a detailed description of how this set of 51 was obtained. These were used to create the tree in **Fig 3.** Sequence identifiers are abbreviated versions of those in **Fig. 3**. The top 43 sequences are putative canonical CBM40 and the bottom 8 *Vibrio*-type. Note the position of the *Actinobacillus* (Gammaproteobacteria; canonical CBM40) sequence, 9th from bottom. Positions of the conserved binding sites in this alignment are as follows (with corresponding positions of the alignment in **Fig. 2** shown in parentheses). The site position common to both canonical and *Vibrio* types is 96 (119 in **Fig. 2**). Canonical-only sites: 78 (104), 102 (125), 104 (127), 116 (135), 118 (137), 228 (226), 241 (233). *Vibrio* type-only sites: 39 (66), 41 (68), 79 (105), 218 (216), 235 (231), 240 (232).

Supplementary Figure 5 Analysis of STD NMR spectra of the binding of (**a**) Neu5Acα2-3Lac (3'SL) and (**b**) Neu5Acα2-6Lac (6'SL) to *Rg*CBM40

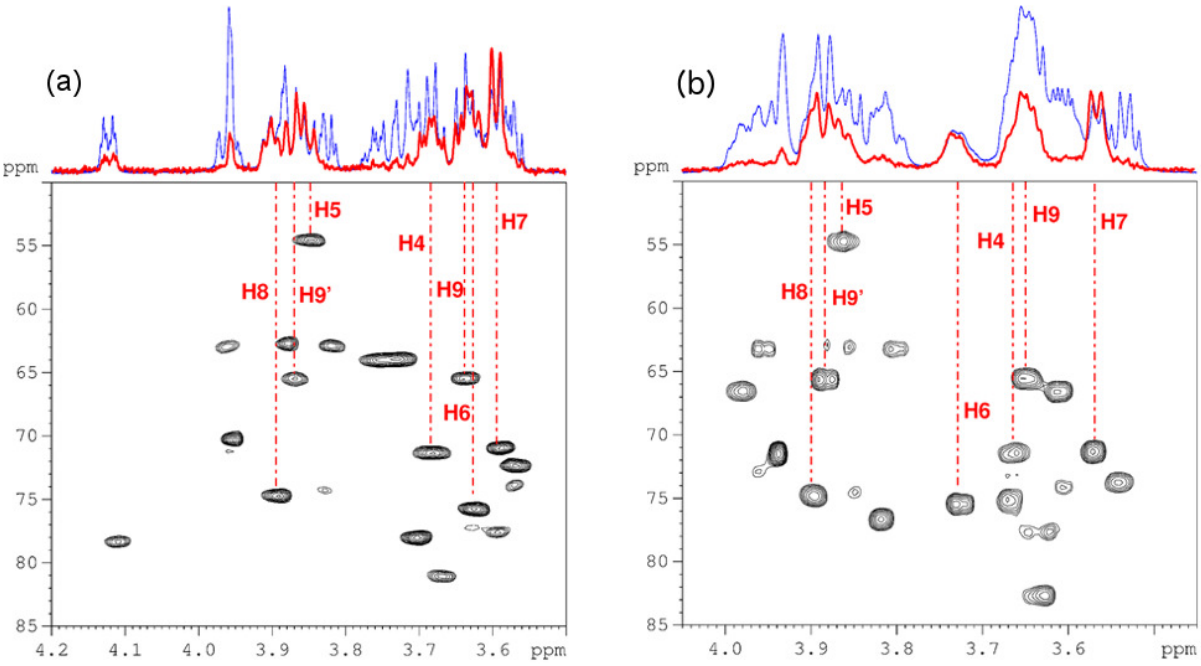

The figure shows ^{1}H,^{13}C HSQC spectra of both ligands and the STD NMR spectra (top, red thick lines) superimposed to the 1D ^{1}H NMR reference spectrum (top, blue thin lines). To facilitate the comparison, the reference spectra are shown at 1/16 of their intensity. HSQC data were fundamental to elucidate the identity of the ligand protons receiving saturation. In both cases, the STD NMR spectra showed that the most intense STD signals corresponded to the protons of the non-reducing terminal sialic acid rings (signals assigned, red dot-dashed line in the HSQC spectra).

Supplementary Figure 6 Binding epitope mapping of sialoglycans bound to *Rg*CBM40 as determined by STD NMR

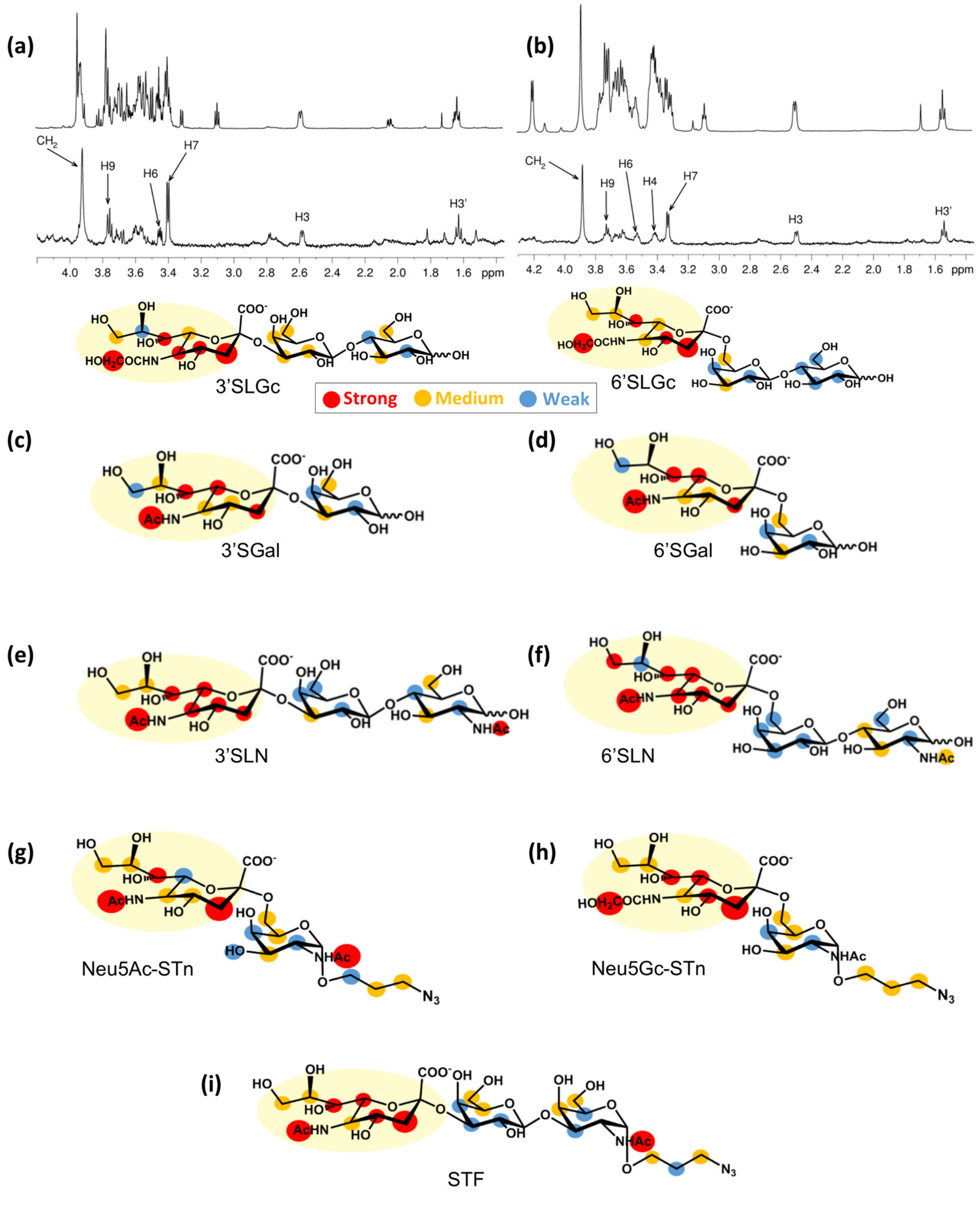

(**a**) Neu5Gcα3Lac (3'SLGc), (**b**) Neu5Gcα6Lac (6'SLGc), (**c**) Neu5Acα3Gal (3'SGal), (**d**) Neu5Acα6Gal (6'SGal), (**e**) Neu5Acα3LacNAc (3'SLN), (**f**) Neu5Acα6LacNAc (6'SLN), (**g**) Neu5Acα6GalαOC$_3$H$_6$N$_3$ (Neu5Ac-STn), (**h**) Neu5Gcα6GalαOC$_3$H$_6$N$_3$ (Neu5Gc-STn), and (**i**) Neu5Acα3Galβ3GalNAcαOC$_3$H$_6$N$_3$ (STF). Legend indicates relative STD intensities normalised at H7: blue, 0–24%; yellow, 25–50%, red 51–100%; larger red dots indicate values over 100%. STD NMR spectra of the binding of 3'SLGc and Neu5Gcα2-6Lac 6'SLGc to *Rg*CBM40 are shown as a representative example.

Supplementary Figure 7 CD spectra of *Rg*CBM40 wt and mutants

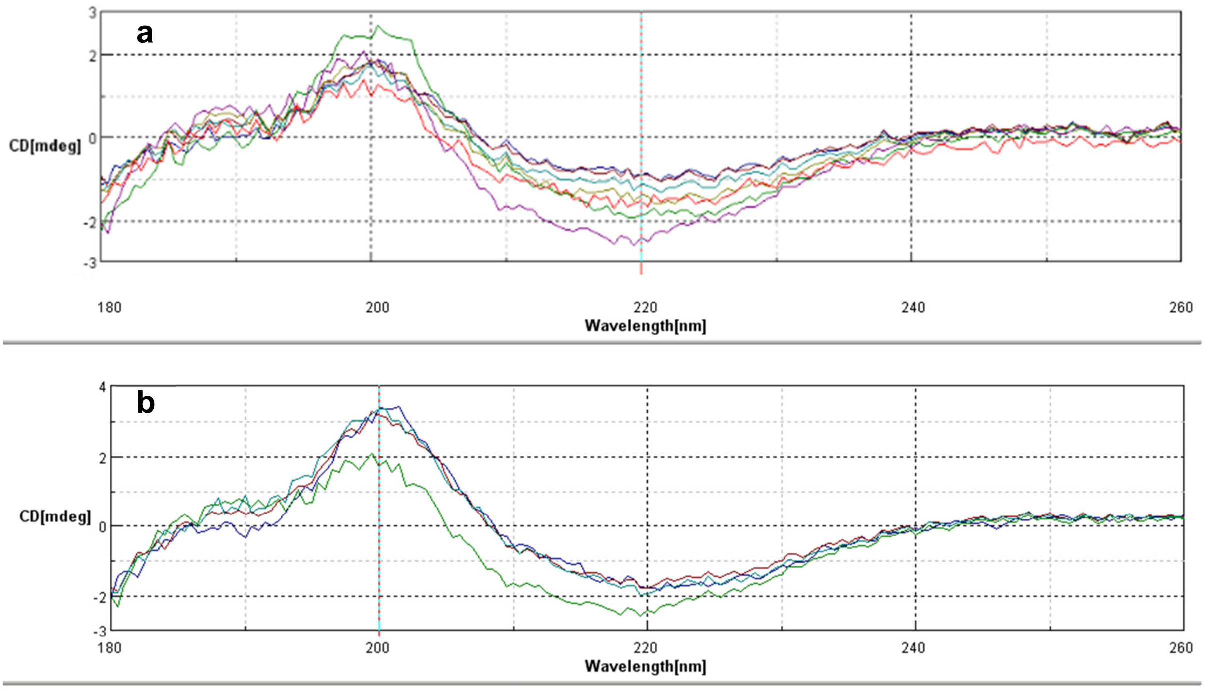

(**a**) WT (light green), I95A (dark purple), Y116A (red), E126A (dark green), R128A (dark blue), Y210A (light blue), boiled WT (light purple). (**b**) WT (dark blue), boiled WT (green), R204A/R128A (brown), R204A-light blue.

Supplementary Figure 8 ITC isotherms of *Rg*CBM40 to sialoglycans

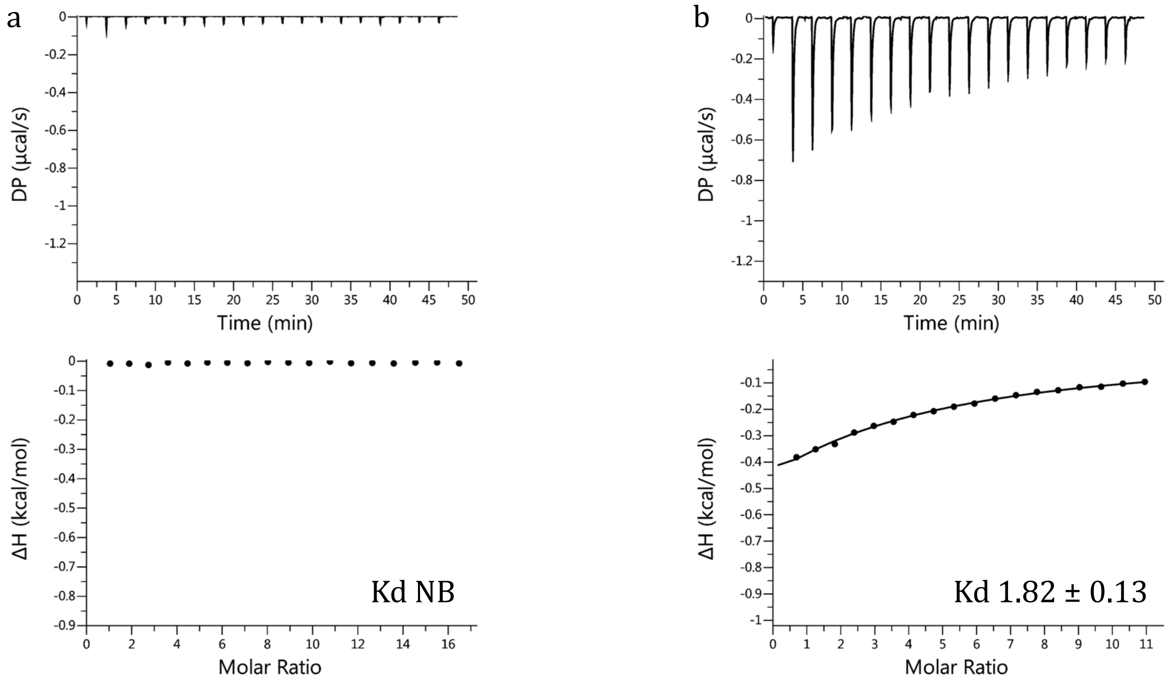

(**a**) *Rg*CBM40 R128A/R204A binding to 3'SL, (**b**) *Rg*CBM40 I95A binding to 3'SL.The Kd is indicated in mM. NB indicates no binding detected. Traces shown are representative examples.

Supplementary Figure 9 Binding epitope mapping of sialoglycans bound to *Rg*CBM40 I95A as determined by STD NMR

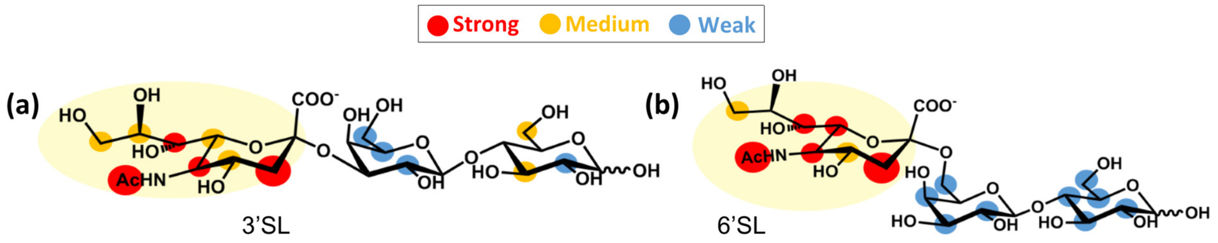

(**a**) 3'SL and (**b**) 6'SL. Legend indicates relative STD intensities normalised at H7: blue, 0–24%; yellow, 25–50%, red 51–100%; larger red dots indicate values over 100%.

Supplementary Figure 10 Substrate specificity of *Rg*NanH and *Rg*GH33 as analysed by HPAEC-PAD

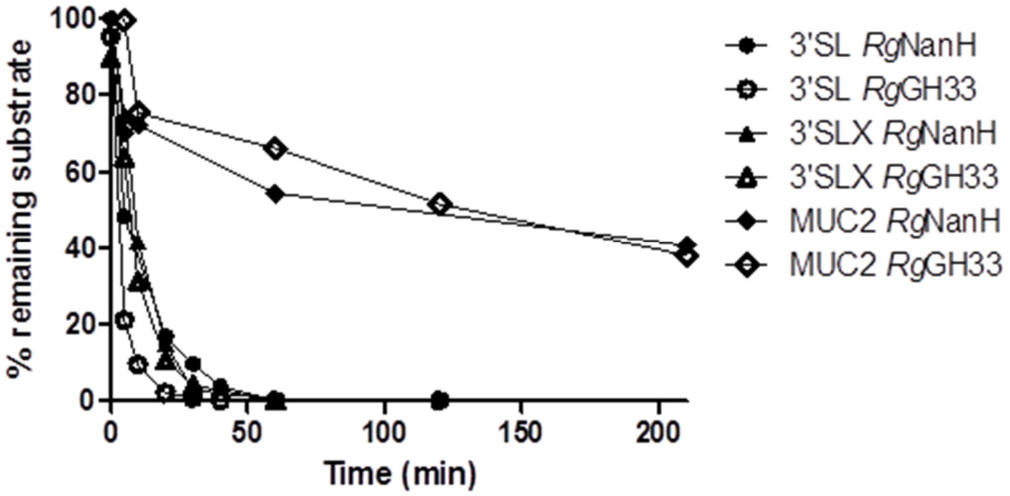

The substrate (α2-3 sialyllactose-3’SL, α2-3 Lewis X-3S’LX or LS174T MUC2-MUC2) was incubated with *Rg*NanH or *Rg*GH33 and the reaction products analysed by HPAEC-PAD. For 3’SL and 3’SLX the % of 3’SL and 3’SLX remaining respectively is plotted. For MUC2, the % of Neu5Ac remaining attached to the MUC2 after removal of the sugars which have been enzymatically liberated is plotted.

Supplementary Figure 11 Immunodetection of IT-sialidase on the cell surface of *R. gnavus* strains

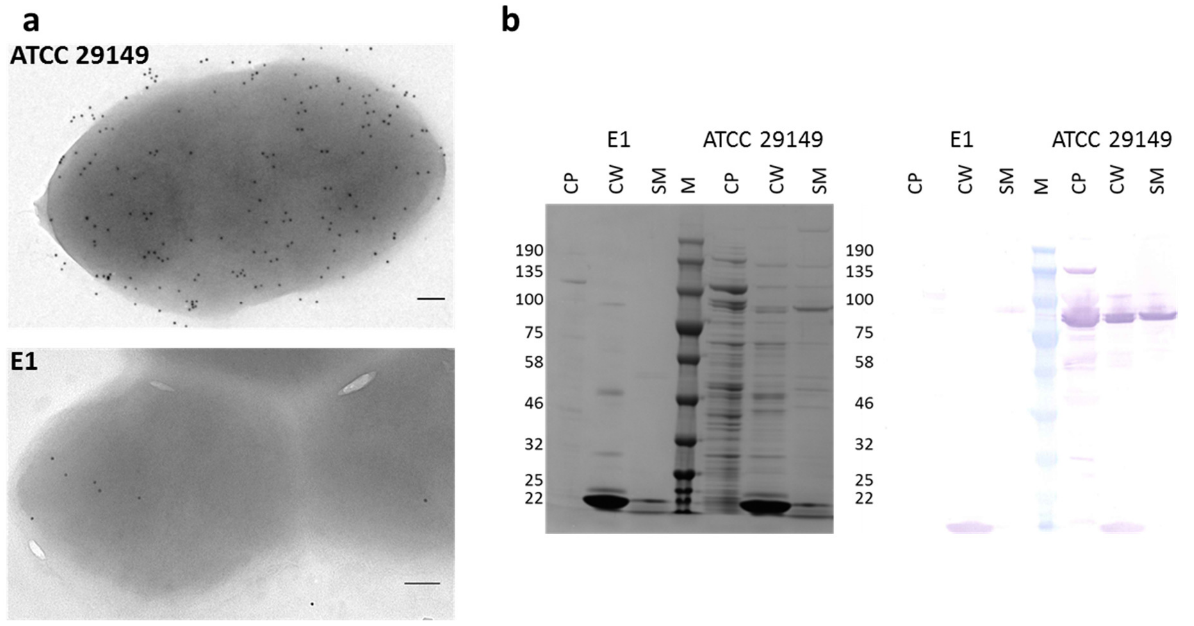

(**a**) Immunogold labelling of *R. gnavus* strains ATCC 29149 and E1. *R. gnavus* strains ATCC 29149 and E1 grown on 3'SL or glucose, respectively, were probed with anti-*Rg*NanH antibody and analysed by transmission electron microscopy (TEM). In each image the scale bar represents 100 nm. (**b**) Western blotting of *R. gnavus* expressed proteins. *R. gnavus* ATCC 29149 and E1 were grown on 3'SL or glucose, respectively. Proteins isolated from the cell wall (CW), cytoplasm (CP), and extracellular proteins from the spent media (SM) were analysed by SDS-PAGE (left) and western blot using anti-*Rg*NanH antibody (right). (M) – Broad Range, Blue Protein Standard (NEB).

Supplementary Figure 12 ELISA of *Rg*CBM40 at different concentrations binding to a range of purified mucins

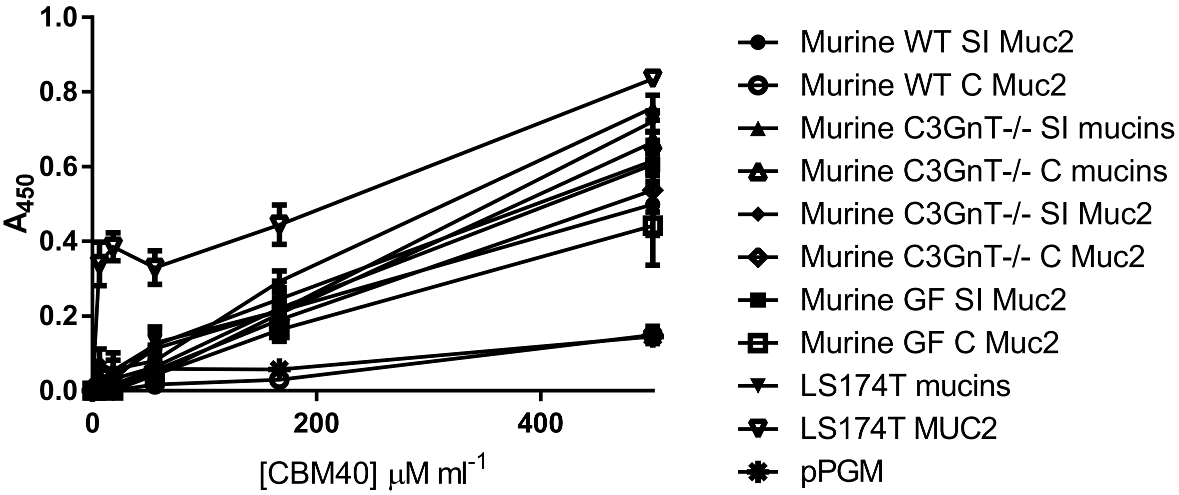

Legend refers to mucin 2 (MUC2) and mixed mucins (mucins) from human cell line LS174T, purified pig gastric mucin (pPGM), mucin 2 (muc2) and mixed mucins from small intestine (SI) and colon (C) of wild type (WT), C3GnT$^{-/-}$ and germ free (GF) mice. The error bars show the standard error of the mean (SEM) of three replicates.

Supplementary Figure 13 A wall-eye stereo image of a portion of the 2Fo-Fc electron density map of the *Rg*CBM40 3’SL complex X-ray crystal structure

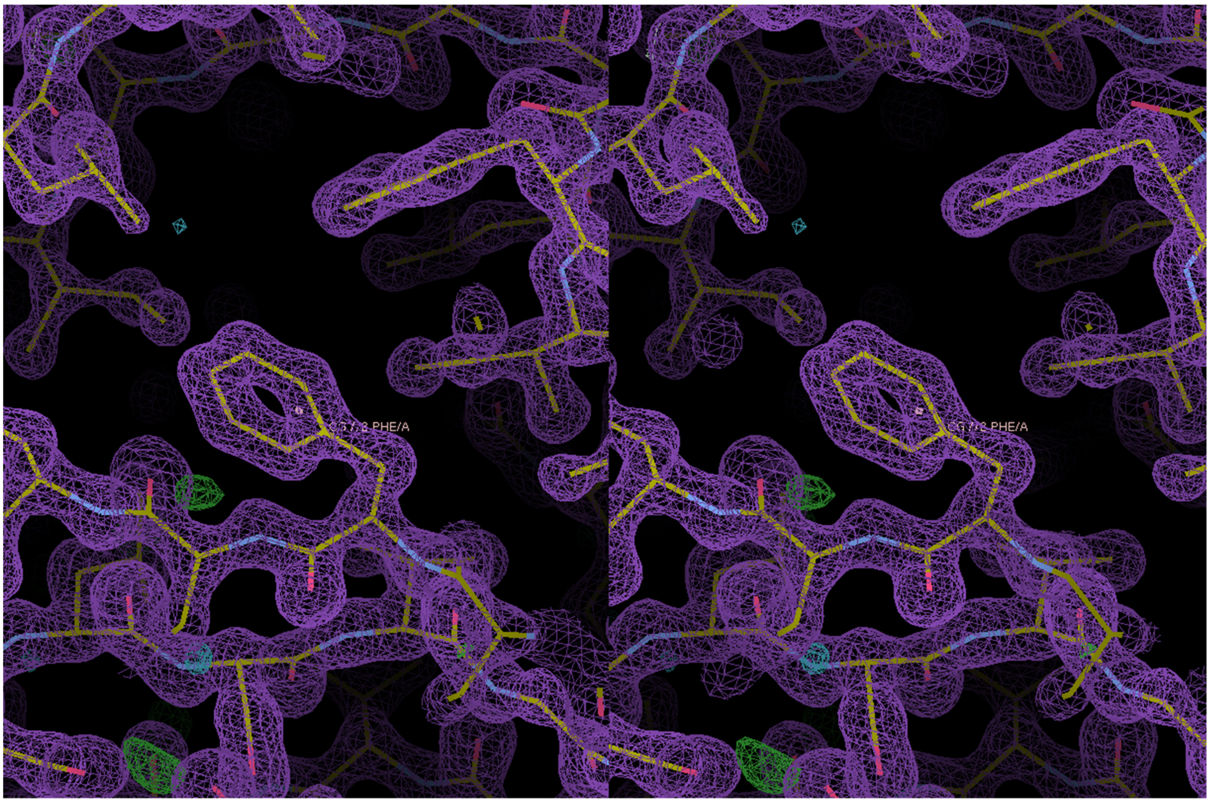

The map is contoured to 2 σ (0.89e/A3) and is centred on Phe72.

Supplementary Figure 14 CBM40 domain sequence alignment used to create the pHMMs

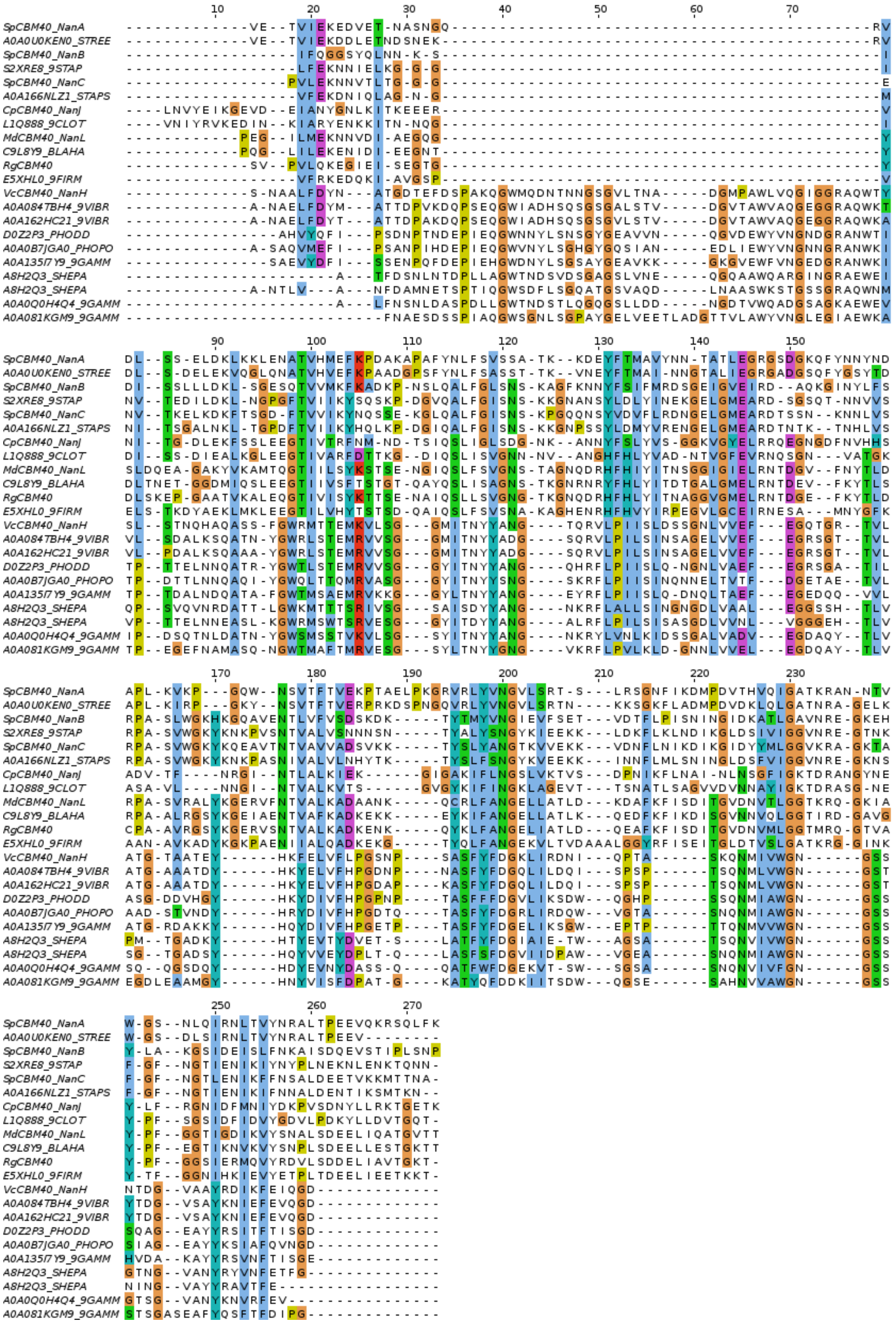

This is based on the structural alignment (see text and **Fig. 2**), with additional homologous segments selected from the result of Blast searches of the Uniprot database. These hits (one for each of the canonical and 9 for the *Vibrio*-type) were selected on the basis of balancing lowest sequence identities possible and a range of taxa while maintaining most or all of the conserved CBM40 features (see **Fig. 2**). Most of the proteins are annotated as 'sialidase'. The 'combined', canonical and *Vibrio*-type pHMMs were constructed using HMMER3 from respectively all, the first 12, and the last 10 sequences. Refer to the text for the key to the 7 original sequences. The remaining 14 sequence names are UniProt identifiers.

Supplementary Tables

Supplementary Table 1 Kd (mM) of *Rg*CBM40 wild type (WT) and mutants for different sugars, as determined by ITC

*Rg*CBM40	Neu5Ac	3'SL	6'SL	Neu5Gc	3'SLGc	6'SLGc
WT	21*	0.57 ± 0.05	1.70 ± 0.14	21*	2.69 ± 0.86	11*
I95A	23*	1.82 ± 0.13	1.37 ± 0.18	NT	NT	NT
Y116A	NB	NB	NB	NT	NT	NT
E126A	NB	NB	NB	NT	NT	NT
R128A	NB	NB	NB	NT	NT	NT
R204A	NB	NB	NB	NT	NT	NT
R128A/R204A	NB	NB	NB	NT	NT	NT
Y210A	NB	NB	NB	NT	NT	NT

The cell contains 115-230 µM protein and the syringe 10 mM sugar (25 mM for Neu5Ac).

The error reported is the standard deviation of three results.

* These values are estimates only as the Kd is too high to determine with the concentration of sugar used.
NB- no binding detected, Kd >>25 mM
NT-not tested

Supplementary Table 2. Thermodynamic parameters for *Rg*CBM40 WT and I95A binding to 3'SL and 6'SL

***Rg*CBM40 + sugar**	**ΔH (kcal/mol)**	**ΔG (kcal/mol)**	**-TΔS (kcal/mol)**
WT + 3'SL	-5.90 ± 0.72	-4.43 ± 0.05	1.47 ± 0.77
I95A +3'SL	-4.71 ± 0.43	-3.74 ± 0.04	0.97 ± 0.47
WT + 6'SL	-9.61 ± 0.44	-3.78 ± 0.05	5.82 ± 0.48
I95A + 6'SL	-3.79 ± 0.24	-3.91 ± 0.07	-0.12 ± 0.3

Enthalpy (ΔH), Gibbs free energy (ΔG) and entropy (-TΔS) values are shown. The error reported is the standard deviation of three results.

Supplementary Table 3. Sialic acid levels in purified mucins as determined by MS

**Mucin**	% sialylated structures
LS174T MUC2	91
LS174T mucins	70
Murine *C3GnT*$^{-/-}$ SI Muc2	59
Murine GF SI Muc2	62
Murine *C3GnT*$^{-/-}$ C Muc2	34
Murine WT SI Muc2	24
Murine GF C Muc2	8
pPGM	2
Murine WT C Muc2	0

The mucins are mucin 2 (MUC2) and mixed mucins (mucins) from human cell line LS174T, purified pig gastric mucin (pPGM), and muc2 from germ free (GF), wild type (WT), and *C3GnT*$^{-/-}$ mutant mice.

Supplementary Table 4. Primer sequences used in this study

Protein encoded	Direction	Sequence (5'-3')
*Rg*NanH WT	F	GATATCGGATCCCAAGAGGCCCAGACAG
	R	TGGTGCTCGAGTTATGGTTGAACTTTCAGTTCATC
*Rg*CBM40 WT	F	GATATCGGATCCGTGTTGCAAAAGGAAGGAATC
	R	GGTGCTCGAGTTACTTTCCTGTCACAGCAATAAG
*Rg*GH33 WT	F	GATATCGGATCCAATATCTTTTATGCAGGAGATGC
	R	TGGTGCTCGAGTTATGGTTGAACTTTCAGTTCATC
*Rg*GH33 D282A	F	GTATTGATCTTACTTTTTGCAGCATGGGTGCCACCATATCTTG
	R	CAAGATATGGTGGCACCCATGCTGCAAAAAGTAAGATCAATAC
*Rg*CBM40 I95A	F	CAACCAGTGAAAATGCGGCTCAATCGTTATTGAGTG
	R	CACTCAATAACGATTGAGCCGCATTTTCACTGGTTG
*Rg*CBM40 Y116A	F	GATAGACATTTCCACTTAGCTATCACAAATGCAGGCGG
	R	CCGCCTGCATTTGTGATAGCTAAGTGGAAATGTCTATC
*Rg*CBM40 E126A	F	GCGGCGTAGGTATGGCATTGAGAAATACAG
	R	CTGTATTTCTCAATGCCATACCTACGCCGC
*Rg*CBM40 R128A	F	GCGTAGGTATGGAATTGGCAAATACAGATGGCGAG
	R	CTCGCCATCTGTATTTGCCAATTCCATACCTACGC
*Rg*CBM40 R204A	F	GTAATGCTGGGCGGTACCATGGCTCAGGGAACCGTTGCCTATC
	R	GATAGGCAACGGTTCCCTGAGCCATGGTACCGCCCAGCATTAC
*Rg*CBM40 R128A/R204A	F (R128A)	GCGTAGGTATGGAATTGGCAAATACAGATGGCGAG
	F (R204A)	GTAATGCTGGGCGGTACCATGGCTCAGGGAACCGTTGCCTATC
	R (R128A)	CTCGCCATCTGTATTTGCCAATTCCATACCTACGC
	R (R204A)	GATAGGCAACGGTTCCCTGAGCCATGGTACCGCCCAGCATTAC
*Rg*CBM40 Y210A	F	CAGGGAACCGTTGCCGCTCCATTTGGAGGTTC
	R	GAACCTCCAAATGGAGCGGCAACGGTTCCCTG

The direction of the primers is either forward (F) or reverse (R).

Supplementary Methods

Construction of the domain sequence profile models representing different types of CBM40 domains, and comparison with existing models. Models (pHMMs) are depicted as truncated boxes. Numbers of hits, including the Venn diagrams, are numbers of domains (not protein sequences) which match each model with an i-Evalue of $\leqslant 10^{-6}$ when searching the 176,818,559 protein sequences obtained from 67,248 prokaryote genomes (NCBI, April 2016), using the HMMER3 software. **(a)** Alignment produced from a manual inspection of the 3D structures of seven CBM40 domains, including *Vc*CBM40 (see main manuscript text), supplemented with a selection of homologous domain sequences from the UniProt database, giving an alignment of 12 canonical and 10 *Vibrio*-type CBM40s (see **Supplementary Fig. 14**). This was also divided into two components (canonical-only and *Vibrio* type-only), and all three were used to produce profile Hidden Markov Models using HMMER3. **(b)** The principal Pfam family matching known canonical CBM40s is "Sialidase, N-terminal domain" (abbreviated in Pfam to "Sialidase" which is potentially confusing, and has therefore been referred here as "Sialidase(NTD)". Furthermore, around half of the Sialidase(NTD)-matching domains also matched a second Pfam family, "Laminin_G_3" (also known as “Concanavalin A-like lectin/glucanases”) - which is a member of the same wider superfamily (clan) as Sialidase(NTD). This should not be confused with proteins having two domains in different parts of the sequence, each matching a different family – which does occur in many cases. **(c)** Our own canonical CBM40 model performs near identically to Sialidase(NTD), while finding a few additional matches. We consider the approximately 16,000 domains matching either canonical CBM40 or Sialidase(NTD) as the 'canonical CBM40 hits', and the approximately 13,000 which match only Laminin_G_3 as 'LG3-only'. **(d)** Unlike the canonical CBM40s, there appeared to be previously no domain model which strongly matches the *Vibrio*-type CBM40. However, some domain hits to our new *Vibrio*-type model also match the Pfam family "Sial-lect-inser" (also known as "*Vibrio cholerae* sialidase, lectin insertion"). Our *Vibrio*-type model captures almost all hits of Sial-lect-inser, as well as many others not matched by it. Analogous to the CBM40/Laminin_G_3 incidence, a number of proteins contained two domains, one matching each of the *Vibrio*-type CBM40 model, and the Sial-lect-inser. **(e)** The new combined CBM40 model succeeds (red ovals) in matching most *Vibrio* type and Sial-lect-inser domains as well most canonical CBM40s, while excluding almost all of the LG3-only domains.

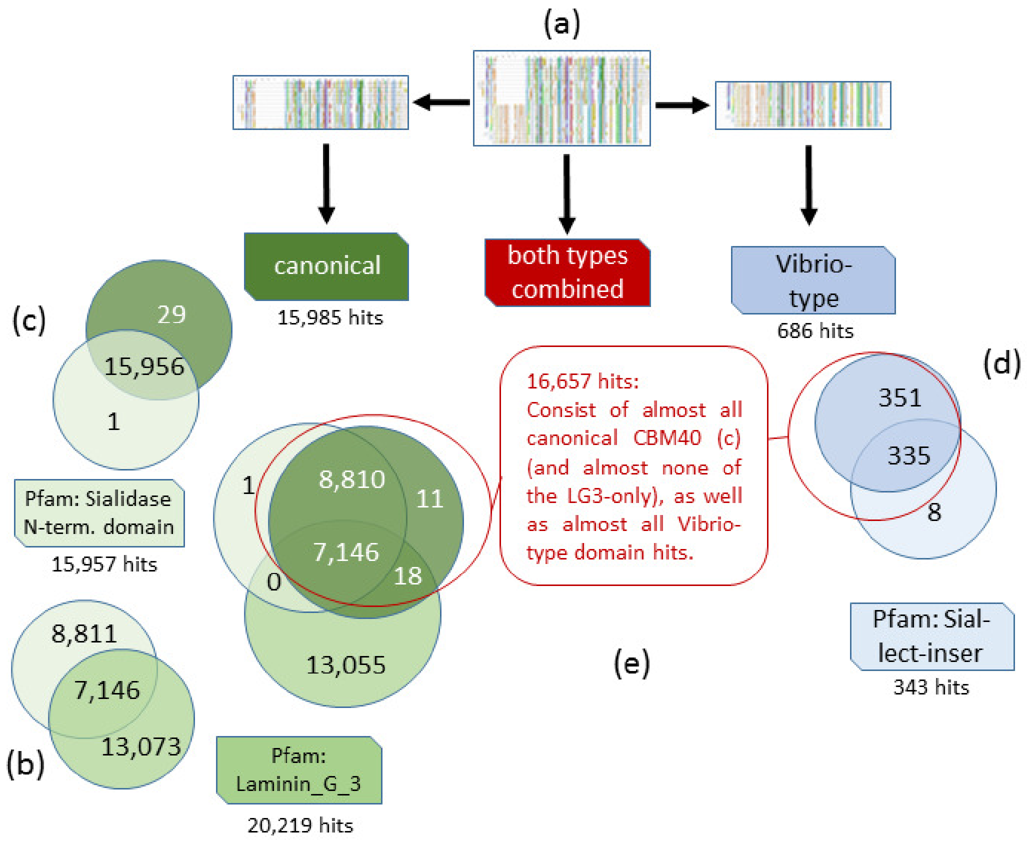

(a)
canonical
15,985 hits
both types combined
Vibrio-type
686 hits
(c)
29
15,956
1
Pfam: Sialidase N-term. domain
15,957 hits
1
8,810
11
7,146
0
18
13,055
16,657 hits: Consist of almost all canonical CBM40 (c) (and almost none of the LG3-only), as well as almost all Vibrio-type domain hits.
(d)
351
335
8
Pfam: Sial-lect-inser
343 hits
(e)
8,811
7,146
13,073
(b)
Pfam: Laminin_G_3
20,219 hits

Full breakdown of the hits of the combined-model. The numbers of domain hits for each pHMM in the database of 176,818,559 protein sequences obtained from 67,248 annotated prokaryote genomes, was determined using HMMER3 (hmmsearch) with a threshold of i-Evalue of $\leqslant 10^{-6}$. See above for details of each model. The numbers of hits which match 1 or more of the models are shown in a single Venn diagram. This illustrates that at this significance cut-off, there was no domain matching both a canonical and *Vibrio* type. However, some combined-model hits (7,146 + 18) matched both the Sialidase(NTD) and Laminin_G_3, which both belong to the same superfamily (see above). Similarly, 331 combined-model hits matched both the *Vibrio*-type CBM40 and Sial-lect-inser. The 16,657 combined-model hits consist of only 396 unique sequences, and these were classified based on the four sub-types (canonical CBM40, Laminin_G_3, Vibrio-type CBM40, Sial-lect-inser), with a manual evaluation of binding sites resolving E-value "ties" or near-ties in a few (18) cases. Discarding the non-CBM40 types resulted in 352 CBM40 hit sequences (canonical and *Vibrio* type), of which 3 were excluded (as representing partial matches to the query pHMM). Application of a redundancy threshold of $\leqslant$ 80% sequence identity (using Jalview[1]) resulted in 51 sequences (**Supplementary Fig. 4**) used for the phylogenetic analysis (**Fig. 3**).

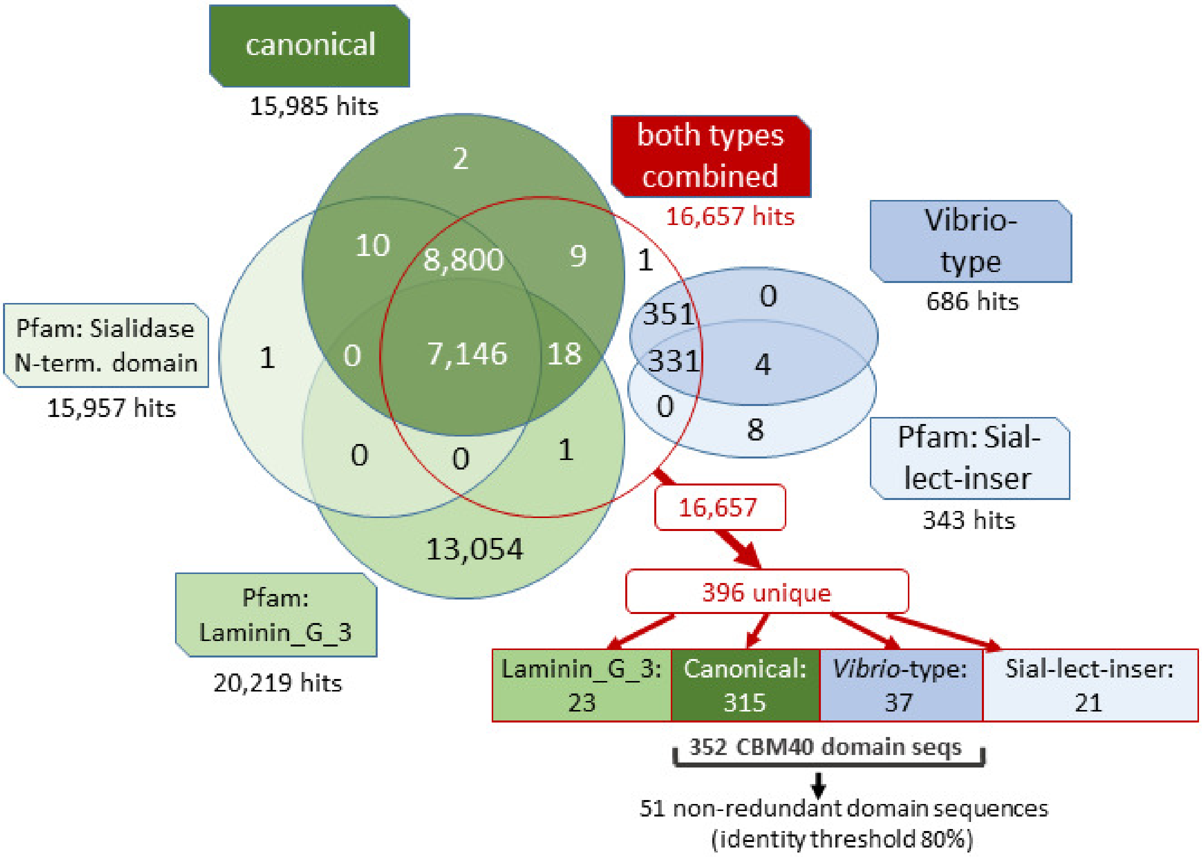

Supplementary reference:

1. Waterhouse, A. M., Procter, J. B., Martin, D. M. A., Clamp, M. & Barton, G. J. Jalview Version 2-a multiple sequence alignment editor and analysis workbench. Bioinformatics 25, 1189-1191 (2009).
